# Stress- and metabolic responses of *Candida albicans* require Tor1 kinase N-terminal HEAT repeats

**DOI:** 10.1371/journal.ppat.1010089

**Published:** 2022-06-10

**Authors:** Wanjun Qi, Maikel Acosta-Zaldivar, Peter R. Flanagan, Ning-Ning Liu, Niketa Jani, José F. Fierro, María T. Andrés, Gary P. Moran, Julia R. Köhler

**Affiliations:** 1 Division of Infectious Diseases, Boston Children’s Hospital/Harvard Medical School, Boston, Massachusetts, United States of America; 2 Division of Oral Biosciences, School of Dental Science, Trinity College Dublin, Ireland; 3 Laboratory of Oral Microbiology, University Clinic of Dentistry (CLUO), and Department of Functional Biology (Microbiology), Faculty of Medicine, University of Oviedo, Oviedo, Asturias, Spain; Yonsei University, REPUBLIC OF KOREA

## Abstract

Whether to commit limited cellular resources toward growth and proliferation, or toward survival and stress responses, is an essential determination made by Target of Rapamycin Complex 1 (TORC1) for a eukaryotic cell in response to favorable or adverse conditions. Loss of TORC1 function is lethal. The TORC1 inhibitor rapamycin that targets the highly conserved Tor kinase domain kills fungal pathogens like *Candida albicans*, but is also severely toxic to human cells. The least conserved region of fungal and human Tor kinases are the N-terminal HEAT domains. We examined the role of the 8 most N-terminal HEAT repeats of *C*. *albicans* Tor1. We compared nutritional- and stress responses of cells that express a message for N-terminally truncated Tor1 from repressible *tetO*, with cells expressing wild type *TOR1* from *tetO* or from the native promoter. Some but not all stress responses were significantly impaired by loss of Tor1 N-terminal HEAT repeats, including those to oxidative-, cell wall-, and heat stress; in contrast, plasma membrane stress and antifungal agents that disrupt plasma membrane function were tolerated by cells lacking this Tor1 region. Translation was inappropriately upregulated during oxidative stress in cells lacking N-terminal Tor1 HEAT repeats despite simultaneously elevated Gcn2 activity, while activation of the oxidative stress response MAP kinase Hog1 was weak. Conversely, these cells were unable to take advantage of favorable nutritional conditions by accelerating their growth. Consuming oxygen more slowly than cells containing wild type *TOR1* alleles during growth in glucose, cells lacking N-terminal Tor1 HEAT repeats additionally were incapable of utilizing non-fermentable carbon sources. They were also hypersensitive to inhibitors of specific complexes within the respiratory electron transport chain, suggesting that inefficient ATP generation and a resulting dearth of nucleotide sugar building blocks for cell wall polysaccharides causes cell wall integrity defects in these mutants. Genome-wide expression analysis of cells lacking N-terminal HEAT repeats showed dysregulation of carbon metabolism, cell wall biosynthetic enzymes, translational machinery biosynthesis, oxidative stress responses, and hyphal- as well as white-opaque cell type-associated genes. Targeting fungal-specific Tor1 N-terminal HEAT repeats with small molecules might selectively abrogate fungal viability, especially when during infection multiple stresses are imposed by the host immune system.

## Author summary

Whether growing harmlessly on our mucous membranes in competition with bacterial multitudes, or invading our tissues and bloodstream, the fungus *Candida albicans* must be capable of rapid growth when it finds abundant nutrients and favorable conditions. It must also be able to switch to stress- and survival mode when encountering host immune cells and when starving for nutrients. Tor1 kinase is the central regulator at the heart of these cellular decisions. As an essential protein, it is an attractive drug target. But the Tor1 kinase domain is very similar to its human counterpart, rendering its inhibitors like rapamycin toxic for humans. We identified a region of helical protein-protein interaction domains, the N-terminal HEAT repeats, as the least conserved part of *C*. *albicans* Tor1. Using genetic- and genome-wide expression analysis, we found that 8 N-terminal HEAT repeats are required for growth acceleration in nutrient-rich environments and for decreased translation in starvation- and stress conditions. This Tor1 region contributes to oxidative-, cell wall- and heat stress reponses, to hyphal growth and to respiration, but apparently not to plasma membrane stress endurance or fermentation. Small molecules that disrupt the protein-protein interactions mediated by this region could become fungal-selective inhibitors of Tor kinase.

## Introduction

The Target of Rapamycin Complex 1 (TORC1) makes fundamental decisions in the life of a eukaryotic cell. It collects information from numerous sources on conditions that affect the cell’s chances of successful growth and proliferation. It then directs downstream regulators to promote anabolic- or stress- and survival responses. Called “the brain of the cell” by Michael Hall [1], TORC1, by extension of this metaphor, integrates data from a plethora of afferent projections in order to send distinct efferent signals to its effectors. In contrast to the brain, in which afferent and efferent fibers can be anatomically separated, TORC1 has only its enormous protein-protein interaction domains to invite upstream signaling molecules to dock and to select its downstream targets for phosphorylation, or to exclude them as appropriate from the kinase catalytic domain. Considering the large number of signaling inputs into TORC1 and its substantial number of effectors, very little is known about where the relevant afferent and efferent elements of the pathway interact with components of the complex.

Appropriate responses to changing favorable or unfavorable environmental conditions are crucial for all free-living microorganisms. Saprophytes like *Saccharomyces cerevisiae* and *Schizosaccharomyces pombe* experience fluctuations of nutrient availability, temperature, osmolarity, oxygen tension and redox potential. Controlled cessation of anabolic activity and regulated exit from the cell cycle as well as induction of stress responses promote survival during adverse conditions, while prompt resumption of translation, organelle biosynthesis and DNA replication enhance competitive fitness in a favorable environment. A commensal and pathogen like *Candida albicans* additionally contends with the density of competing flora in the host gastrointestinal tract, and with stresses actively imposed by the host immune system. These stresses include membrane stress induced by antimicrobial peptides, oxidative- and cell wall stress generated by host phagocytes and heat stress through fever as a host defense.

TORC1 was discovered through genetic analysis of rapamycin’s mechanism of action [2], a streptomycetal secondary metabolite originally isolated for its antifungal activity against *C*. *albicans* [3,4]. Found to be toxic for human cells, especially for lymphocytes whose protein production and proliferation are integral to their function, rapamycin’s development as an antifungal agent was abandoned [5]. The discovery of its target over a decade later [2] set in motion a plethora of novel areas of investigation now known to be central to eukaryotic cell biology. Given rapamycin’s cidal potency against *C*. *albicans* and other fungi [6,7], as well as against other pathogens relying on eukaryotic mechanisms of growth and proliferation control [8], inhibitors with specificity for the fungal-, and neutrality toward the human TORC1 could be of great therapeutic interest [6]. In order to develop such compounds, more detailed understanding of the functional role of each of the Tor kinase protein domains and their subsegments, as well as of the structural differences between fungal and mammalian TORC1, will be a precondition.

In *S*. *cerevisiae*, TORC1 is a dimer of each of the components, the kinases Tor1 or Tor2, the TORC1-defining component Kontroller of Growth (Kog1), the WD40 repeat-containing regulator and stabilizer of the TORC1 complex, Lethal with Sec13 protein 8 (Lst8) [9], and Tor Complex One 89 kD protein (Tco89), noted for its role in cell wall integrity [10] [11]. *C*. *albicans*, like mammals, has only one Tor kinase, Tor1. Homologs of the other *S*. *cerevisiae* TORC1 components are also present in *C*. *albicans*.

Tor kinase is a serine/threonine protein kinase despite its homology to phosphatidylinositol 3-kinases. Its domain structure, consisting principally of protein-protein interaction domains, is conserved to a large extent across eukaryotes, so that fungal, mammalian and plant structures have been used to analyze the functional units [12]. Its C-terminal kinase domain consists of an N-terminal and a C-terminal lobe that together form a deep catalytic cleft which enables inhibition by blocking substrate access [13]. At the N-terminus of Tor kinase are two domains of helical repeats, the N-terminal and the middle HEAT (huntingtin, elongation factor 3, a subunit of PP2A, and TOR1) domains, followed by the FAT (named for FKBP12 Rapamycin Associated Protein (FRAP), ATM, TRRAP) domain. The FRB (FKBP12-Rapamycin binding) domain is part of the N-terminal lobe of the kinase domain, and in mTOR acts as a gatekeeper to control substrate access to the catalytic site [13]. At its C-terminus the kinase domain is flanked by C-terminal FAT domains [14].

N terminal HEAT repeats consist of arrays of two antiparallel alpha helices of 10–20 amino acid residues’ length each separated by intraunit loops of 5–8 residues [15]. *S*. *cerevisiae* Tor kinase N-terminal HEAT repeats were shown by electron microscopy to interact closely with the C-terminus of Kog1 [16], a scaffold for interacting proteins and a gatekeeper for substrates to the kinase active site [17]. A cryo-EM structure of Tor kinase complexed with Lst8 of the yeast *Kluyveromyces marxianus* showed its N terminus to consist of a curved “spiral” of 36 helices comprising ~800 residues, representing the N-terminal HEAT domain, followed by a linker that connects to another “bridge” sequence of 14 helices comprising ~400 residues, representing the middle HEAT domain [18]. The major interaction sites between two KmTor kinase molecules in the dyadic complex described by these authors are formed reciprocally between the N-terminal (spiral) and middle (bridge) HEAT domains of the two Tor kinase molecules which connect in a yin-yang orientation. In their projection, Kog1 binds to the N-terminal and middle HEAT domain of the opposite Tor kinase subunit in the complex, contributing to the stability of the dimer [18].

The N-terminal HEAT domain of mammalian Tor kinase is predicted to be exposed at the surface of mTORC1 and bind its regulators [17]. As deduced from aligning the CaTor1 sequence with mTOR, whose HEAT repeats include the N-terminal 1382 amino acid residues [19], CaTor1 HEAT repeats comprise 1334 N-terminal amino acids. N-terminal truncation of mTOR by 297 residues weakens its interaction with the conserved Kog1 homolog Raptor [20].

An N-terminally truncated human mTOR consisting only of residues 1376 to 2549, in complex with full-length human mLST8, was able to phosphorylate a classic TORC1 substrate, S6 kinase1 (S6K1), in vitro [13], highlighting that Tor kinase HEAT repeats and Kog1 are not necessary for catalytic activity; they serve to recruit substrates for, and to regulate the kinase. Recent structural analysis of mTOR has shown that binding of its activator Rheb induces conformational changes involving the N-HEAT domain and the FAT domain that realign active-site residues in the catalytic cleft to facilitate the enzymatic reaction [12]. It is unclear whether this allosteric regulatory mechanism is conserved in model fungi. In *S*. *cerevisiae* the Rheb homolog Rhb1 acts on arginine uptake and is not essential [21] whereas in *S*. *pombe* it is essential and acts in both basic amino acid uptake and TORC1-controlled processes like growth, differentiation for mating and cell cycle exit [22]. In *C*. *albicans*, Rhb1 and its upstream regulator Tsc2 are involved in rapamycin susceptibility and regulate filamentous growth and expression of the ammonium transporter Mep2 and the secreted proteinase Sap2 [23,24]. CaRhb1 is required for normal cell wall stress resistance and cell wall integrity signaling [23]. It is required for appropriate Rps6 phosphorylation, a downstream effect of active TORC1 [25]; deletion mutants in *RHB1* differentially transcribe genes controlled by TORC1 like those involved in ribosome biogenesis and amino acid biosynthesis [24, 26]. Rhb1 hence is a component of the *C*. *albicans* TORC1 signaling pathway but details of its physical interaction with Tor1, if any, are unknown.

Inhibition of mTor kinase by PRAS40 involves its binding to the FRB domain and obstructing the catalytic cleft [12]. This regulatory mechanism is not present in yeast or *C*. *albicans* which do not have a PRAS40 homolog. Other structural analyses of mTor kinase, including its interactions with the major substrates 4EBP1 and S6K1, similarly cannot be extrapolated to the function of *S*. *cerevisiae* and *C*. *albicans* TORC1, since regulating translation initiation does not involve a 4EBP1 homolog in these fungi and the orthologous role of Sch9 as an S6K1 homolog is still debated [27, 28]. Therefore, despite higher-level structural similarities between fungal and mammalian TORC1, Tor kinase activity is regulated differently in fungi than in mammals and the roles of its distinct domains and their physical interaction partners are not understood in detail.

The N-termini of fungal and human Tor kinase are their most widely divergent domains, suggesting they may be sufficiently structurally and functionally distinct that selective chemical targeting could be achievable. The large size and complexity of the N- and M-HEAT domains [18] indicates commensurate complexity of interactions. A functional dissection of their individual roles may aid in further characterizing afferent as well as efferent signal fluxes of Tor1. Responses to nitrogen source quality and quantity and control of protein synthesis are central to TORC1 signaling [29–31], as recently reviewed e.g. in [32]. In *S*. *cerevisiae* and *C*. *albicans*, TORC1 responds to carbon source quality and quantity [31] and it controls carbon source-acquisition and -metabolism genes of *S*. *cerevisiae* [30]. Phosphate repletion is an afferent signal to TORC1 in *S*. *cerevisiae* and *C*. *albicans* and TORC1 activity also affects the *C*. *albicans* phosphate acquisition system, the PHO regulon [33].

As mentioned above, *C*. *albicans* must adapt to rapidly shifting nutritional states as a commensal in the human gastrointestinal tract, and to stresses imposed not only by competing flora but also by the human immune system during commensalism, mucosal- and invasive infection. To maximize fitness in these quickly changing environments, TORC1 of *C*. *albicans* must be finely tunable to an array of environmental parameters in widely varying combinations. The challenges of adapting to varying host environments and the importance of Target of Rapamycin complexes in meeting these challenges are exemplified by the sleeping sickness parasite *Trypanosoma brucei*, whose complex life cycle comprising four cell types alternates between its fly vector and its human host [34]: *T*. *brucei* has four TOR complexes [35,36]. *C*. *albicans* TORC1 is involved in hyphal growth and adhesion [37–40], biofilm formation [26], secretion of aspartyl protease [24], and it responds to nitrogen-, carbon-source [25, 41] and phosphate [33] availability. TORC1 is predicted to be required for *C*. *albicans* fitness in favorable conditions like repletion of distinct nutrients, as well as in manifold stress conditions encountered in the host.

Given the wide array of stimuli to which TORC1 responds and the broad spectrum of responses it controls, we set out to examine by a genetic approach the processes generally regulated by Tor1, and those regulated by the most N-terminal segment of the protein in *C*. *albicans*. We constructed two mutant genotypes in which the single remaining *TOR1* alleles were placed under the control of a repressible regulatory element: wild type full-length *TOR1* or a 5’-truncated *TOR1* allele whose predicted protein product lacks the 8 most N-terminal HEAT repeats. Cells in which these *TOR1* alleles were overexpressed and partially or fully repressed were examined for their responses to nutritional repletion or starvation, and to stresses to which *C*. *albicans* is exposed in the host.

## Results

### Repression of TOR1 transcription decreased growth in a dose-dependent manner

In order to test the role of *C*. *albicans* Tor1 and of its N-terminal HEAT repeats in the response to distinct nutrients and stressors, we constructed strains in which transcription of a single *TOR1* allele is regulated by repressible *tetO*. We deleted the 3’ 6.3 kb of *TOR1* including the kinase domain in one allele to generate several independent heterozygotes. We mutated the second *TOR1* allele in three independent heterozygous deletion mutants in order to detect artifacts arising from suppressor mutations; strains were constructed to express either full-length Tor1 (Tor1-FL) or an N-terminally truncated Tor1 whose Start methionine is residue 382 of the full length protein (Tor1-Del381). In this way, we were able to examine effects of loss of *TOR1* and compare them to specific loss of N-terminal HEAT repeats. In the absence of the repressing compound doxycycline, transcription from this construct is known to be high [42].

Tor kinase N termini consist of arrays of HEAT repeats [15]. Aligning amino acid sequences of *C*. *albicans* Tor1 and the *K*. *marxianus* Tor kinase, whose structure has been elucidated in detail [18], with human mTOR, we noted that the most divergent regions of these orthologs are in their N-termini while their catalytic domains are highly conserved (Table 1 and Fig A in S1 Text). The deleted residues comprise the most divergent regions of the *C*. *albicans* and human Tor orthologs (Table 1 and Fig A in S1 Text); we estimated that phenotype analysis might be able to ascribe specific functionalities to a truncated region of limited extent like the first 8 HEAT repeats, while truncating a larger region could result in global non-specific defects. We used the descriptor *TOR1-Del381* for this *TOR1* allele as well, for the sake of simplicity, though the number 381 refers to the truncated amino acid residues and not to the deleted nucleotides. The mutant protein lacks the 8 N-terminal HEAT repeats, truncating the N-terminal HEAT domain, a predicted interaction site with the regulator Kog1. We chose at least 2 strains from each genotype lineage whose phenotypes were indistinguishable for further analysis of growth- and stress phenotypes. We also used two isogenic wild type strains whose deleted *ARG4* locus was independently restored to one wild type allele (resulting in *ARG4/arg4*). To simplify strain descriptors going forward, we designated as “Del381” or “FL” those strains in which the 5’-truncated *TOR1* allele (encoding an N-terminally truncated protein) or the wild type *TOR1* gene, respectively, were controlled by *tetO*; their respective genotypes were *tor1/tetO-TOR1-Del381* or *tor1/tetO-TOR1*.

**Table 1:** Sequence alignments of *C*. *albicans* and human Tor kinases.

Region	Ca.Tor1 8 N-terminal HEAT repeats	N-HEAT	Middle HEAT	FAT	FRB	Kinase Domain
**Identity**	123/479 (25.7%)	287/932 (30.8%)	116/281 (41.3%)	293/674 (43.5%)	50/96 (52.1%)	315/559 (56.4%)
**Similarity**	213/479 (44.5%)	453/932 (48.6%)	199/281 (70.8%)	409/674 (60.7%)	74/96 (77.1%)	401/559 (71.7%)
**Gaps**	130/479 (27.1%)	182/932 (19.5%)	5/281 (1.8%)	53/674 (7.9%)	0/96 (0.0%)	42/559 (7.5%)
**Aa residues Ca.Tor1**	1–381	22–853	909–1187	1310–1951	1972–2067	1952–2481
**Aa residues Hs.mTor**	1–447	25–874	942–1219	1350–2002	2023–2118	2003–2548

Individual regions of Tor kinase from *C*. *albicans* (Ca.Tor1) and human (Hs.mTOR) were aligned and compared using EMBOSS Needle Pairwise Sequence Alignment (https://www.ebi.ac.uk/Tools/psa/emboss_needle/). Boundaries of each region were chosen according to the amino acid sequence alignment in Fig A of S1 Text.

**Fig 1:**
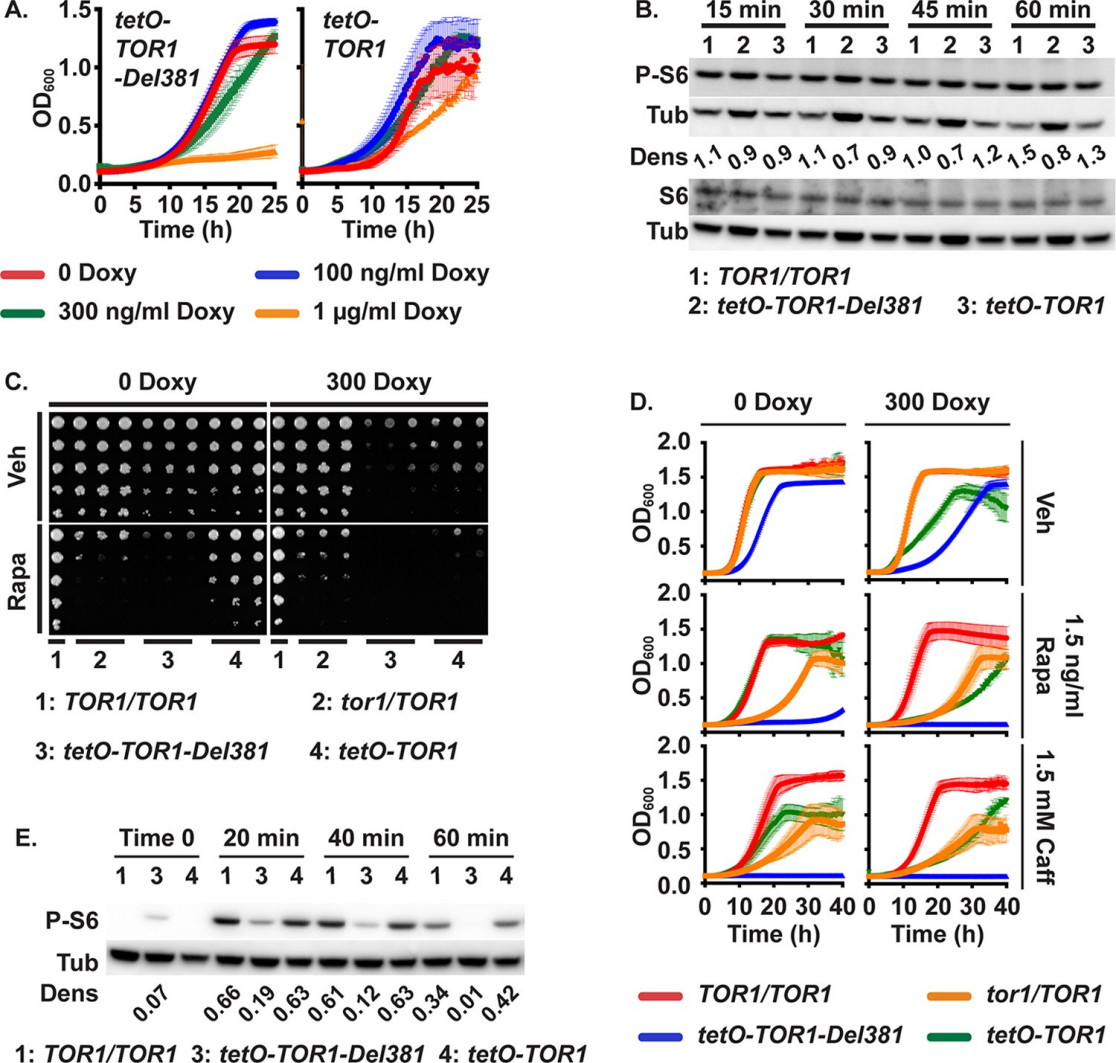
Cells lacking Tor1 N-terminal HEAT repeats were rapamycin hypersensitive. **A.** Cells expressing *TOR1-Del381 or TOR1-FL* from *tetO* were pre-grown in YPD medium for 4 h and inoculated in YPD with increasing concentrations of doxycycline (Doxy). OD_600_ was read every 15 minutes. **B.** Western blot of cells of indicated *TOR1* genotypes, wild type (*TOR1/TOR1*), Del381 and FL, grown in YPD with 5 ng/ml doxycycline for 3.5 h, then inoculated into YPD with 5 ng/ml doxycycline and incubated for indicated time periods (15 min, 30min, 45min, 60min); protein extracts probed with antibody to phosphorylated Rps6 (P-S6), total Rps6 (S6) and tubulin (Tub) as loading control. Dens: signal intensity ratio of P-S6 to Tub. (*TOR1/TOR1*, JKC1713; *tetO-TOR1-Del381*, JKC1441; *tetO-TOR1*, JKC1549). The same samples were run on separate gels to detect either P-S6 or S6. **C.** Dilutions of cells of indicated genotypes were spotted on YPD medium containing vehicle (Veh, 90% ethanol) or 1.5 ng/ml rapamycin (Rapa), without or with 300 ng/ml doxycycline and incubated at 30^o^ for 2 days. (*TOR1/TOR1*, JKC1713; *tor1/TOR1*, JKC1345, JKC1346, JKC1347; *tetO-TOR1-Del381*, JKC1442, JKC1445, JKC1441; *tetO-TOR1*, JKC1543, JKC1546, JKC1549). **D.** Cells of indicated genotypes were grown in YPD medium containing Vehicle (Veh, 90% ethanol), 1.5 ng/ml Rapamycin (Rapa) or 1.5 mM Caffeine (Caff), without or with 300 ng/ml doxycycline. (*TOR1/TOR1*, JKC1713; *tor1/TOR1*, JKC1347; *tetO-TOR1-Del381*, JKC1441; *tetO-TOR1*, JKC1549). **E.** Cells of indicated genotypes were pre-grown in YPD medium with 5 ng/ml doxycycline for 3.5 h (Time 0) and then diluted into fresh YPD medium with 5 ng/ml doxycycline and 25 ng/ml rapamycin. Strains, antibodies and densitometry as in B.

In cells of both mutant genotypes, growth in rich complex medium, YPD, decreased in a dose-dependent manner as concentrations of doxycycline increased. When *TOR1* expression was fully repressed at high concentrations of doxycycline, 1 or 2 μg/ml (Fig B in S1 Text, see time point 0), growth of cells containing the truncated allele was nearly abolished (Fig 1A). Modest growth defects were noted at 300 ng/ml doxycycline in both mutant strains and we consequently chose this concentration for most experiments to examine partial loss-of-function phenotypes (Fig 1A). Apparent residual expression of full-length *TOR1* was sufficient to permit substantial growth during full *tetO* repression with 1 μg/ml doxycycline (Fig 1A), possibly due to inevitable leakiness of an inhibited *tetO* construct, while as noted full repression of *tetO-TOR1-Del381* led to a severe growth defect. Quantitative RT-PCR showed rapid overexpression of *TOR1-Del381* alleles from *tetO* in the absence of doxycycline, while *TOR1-FL* mRNA levels recovered more slowly after *tetO* repression, hinting at a possible role of N-terminal HEAT repeats in the half-life of the *TOR1* mRNA itself (Fig B in S1 Text).

**Fig 2:**
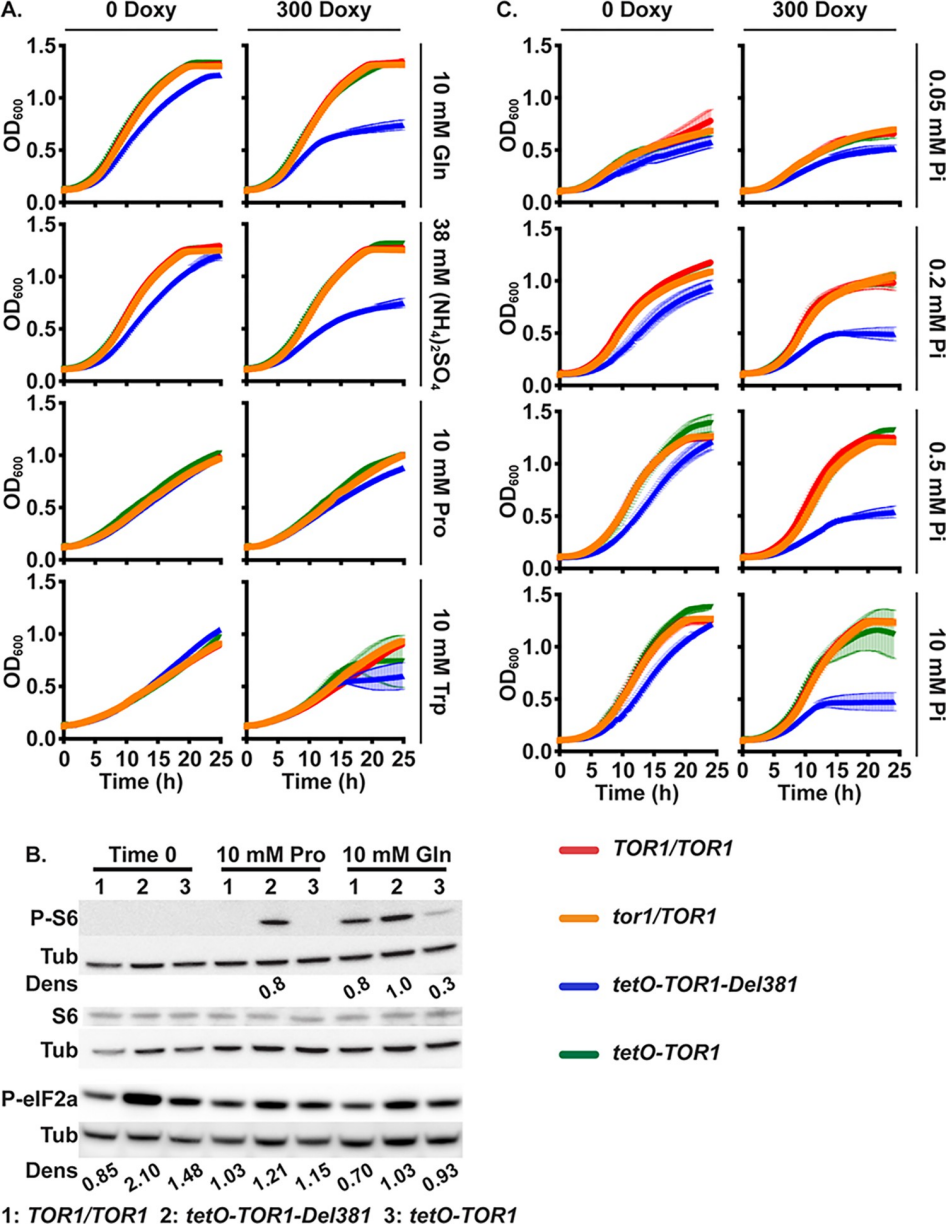
Tor1 N-terminal HEAT repeats were required to accelerate growth and activate TORC1 in response to preferred nitrogen sources and to phosphate. **A.** Cells of indicated genotypes were grown in YNB without ammonium sulfate ((NH_4_)_2_SO_4_) supplemented with 10 mM Glutamine (Gln), 38 mM (NH_4_)_2_SO_4_, 10 mM Proline (Pro) or 10 mM Tryptophan (Trp) as sole nitrogen source, without or with 300 ng/ml doxycycline (300 Doxy). (*TOR1/TOR1*, JKC1713; *tor1/TOR1*, JKC1347; *tetO-TOR1-Del381*, JKC1441; *tetO-TOR1*, JKC1549) **B.** Western blot of cells pre-grown in YPD medium with 2 μg/ml doxycycline for 4 h (Time 0), then incubated in YNB without doxycycline, without (NH_4_)_2_SO_4_, with 10 mM Proline (Pro) or Glutamine (Gln) as sole nitrogen source for 45 min; protein extracts probed with antibody to phosphorylated Rps6 (P-S6), total Rps6 (S6) or phosphorylated eukaryotic translation initiation factor 2A (P-eIF2a), and tubulin (Tub) as loading control. Dens: signal intensity ratio of P-S6 or P-eIF2a to Tub. Strains as in panel B and *TOR1/TOR1*, JKC1361. Same samples were run on separate gels to detect either P-S6 or P-eIF2a. **C.** Cells pre-grown as in A were inoculated into YNB without phosphate supplemented with varying concentration of KH_2_PO_4_ (Pi), without or with 300 ng/ml doxycycline (300 Doxy). Error bars show SD of 3 technical replicates. Strains as in panel A and *tor1/TOR1*, JKC1347.

We assayed TORC1 activity during exponential growth in YPD by the phosphorylation state of ribosomal protein S6 (P-S6) [25], using a minimal doxycycline concentration of 5 ng/ml as a ceiling for the expression level. Cells overexpressing full-length *TOR1* or *TOR1-Del381* from *tetO* showed comparable P-S6 signal intensity to wild type cells in rich medium (Fig 1B), with a slightly weaker P-S6 signal in Del381 cells under these conditions of optimal growth. Levels of total Rps6 protein did not change under any of the experimental conditions we examined and hence are shown only twice (Figs 1B and 2B). We concluded that overexpression of *TOR1* from unrepressed *tetO* does not per se increase immediately measurable TORC1 signaling, possibly because the other TORC1 components required by the stoichiometry of the complex are not available at higher levels.

### Lack of N-terminal HEAT repeats sensitized cells to rapamycin

Since Del381 cells’ growth defect at repressive doxycycline concentrations (1 μg/ml) was more severe than that of FL cells, we examined their response to direct inhibition of Tor1 catalytic activity with rapamycin. Del381 cells were hypersensitive to rapamycin on solid and in liquid medium compared with cells carrying all other *TOR1* alleles, whether or not *tetO* was repressed (Fig 1C and 1D). Rps6 phosphorylation was weaker during rapamycin exposure in Del381 cells at every time point we examined (Fig 1E), indicating rapamycin hypersensitivity of anabolic TORC1 signaling in these cells, possibly due to distortion of the FRB site at which the complex of rapamycin and FKBP-12 docks onto Tor1 and obstructs substrate access to the catalytic cleft.

Caffeine inhibits Tor1 in a manner that is distinct from rapamycin’s mechanism of action but that appears to also involve the FRB domain [43]; in vitro caffeine inhibits ScTor kinase activity at an IC_50_ of 0.28 mM. We examined responses of Del381 and FL cells to caffeine exposure, in comparison with wild type and *tor1/TOR1* heterozygous cells. In contrast to cells bearing the other 3 *TOR1* genotypes, Del381 cells were severely hypersensitive to caffeine whether their *TOR1-Del381* allele was overexpressed or repressed from *tetO* (Fig 1D and 1B in S1 Text). Together, these findings are consistent with the idea that Tor1 FRB domain function is affected when the N-terminal HEAT repeats are absent, though other mechanisms are possible.

### Tor1 N-terminal HEAT repeats were required to accelerate growth in preferred- and to decrease TORC1 signaling in poor nitrogen sources

Preferred nitrogen sources activate *S*. *cerevisiae* TORC1 [44–46], and this effect is conserved in *C*. *albicans* [25]. To test the role of *C*. *albicans* Tor1 and its N-terminal HEAT repeats in cells’ responses to rich versus non-preferred nitrogen sources, wild type, *tor1/TOR1*, Del381 and FL cells were grown in different nitrogen sources. FL cells grew as well as wild type or *tor1/TOR1* heterozygous cells in synthetic YNB medium with 2 preferred (glutamine, ammonium sulfate) and 2 non-preferred (proline, tryptophan) nitrogen sources when *tetO* was derepressed or partially repressed with 300 ng/ml doxycycline (Fig 2A). Del381 cells grew more slowly than wild type or heterozygotes in preferred nitrogen sources known to induce TORC1 signaling [25], ammonium sulfate or glutamine (Fig 2A). They had no specific growth defect in liquid media containing the non-preferred nitrogen sources proline or tryptophan [25] (Fig 2A). In contrast, cells of all genotypes containing a full length *TOR1* allele grew more slowly in non-preferred than in preferred nitrogen sources (Fig 2A). The hypomorphic Del381 phenotype on solid media during partial *tetO* repression with 300 ng/ml doxycycline showed sharply diminished growth on the 4 nitrogen sources tested (Fig B in S1 Text); it was therefore in liquid media that a specificity of Del381 cells’ growth defect for preferred nitrogen sources became apparent. Tor1 N-terminal HEAT repeats were hence specifically required in liquid medium for cells’ growth acceleration during use of preferred nitrogen sources.

To examine the role of Tor1 N-terminal HEAT repeats in TORC1 signaling during growth in distinct nitrogen sources, we assayed TORC1 activity by the P-S6 signal [25]. Expression of *TOR1* was repressed for 4 hours by exposure to 2 μg/ml doxycycline and then released for 45 minutes in media without doxycycline containing glutamine or proline, respectively. Cell lysates were probed with antibody to P-S6 and total Rps6. Del381 cells showed significantly elevated P-S6 signals above those of wild type or FL cells in both conditions: in proline, where wild type and FL cells’ P-S6 signal was undetectable while that of Del381 cells was strong, and in glutamine, where the P-S6 signal from Del381 cells was more intense than that of the other two strains (Fig 2B). Hence, adaptation of TORC1 signaling in response to quality and quantity of nitrogen sources required the complete Tor1 N-terminal HEAT domain.

Eukaryotic initiation factor 2 mediates translational control in response to starvation and environmental stresses in *S*. *cerevisiae* as reviewed e.g. in [47]. During starvation for preferred nitrogen sources and for glucose, the eIF2 alpha subunit (eIF2α) is phosphorylated by the kinase Gcn2 [48] and translation of many anabolic messenger RNAs is inhibited. Using an antibody against the conserved phospho-serine 51 of human eIF2α, we examined whether inappropriately increased TORC1 signaling in Del381 cells might correspond to inappropriately weak translation inhibition signaling through Gcn2, as assayed by eIF2α phosphorylation. To the contrary, we found that eIF2α phosphorylation was increased in Del381 cells (Fig 2B), reflecting increased inhibitory signaling by Gcn2 and suggesting that TORC1- and Gcn2-signaling can become uncoupled when TORC1 lacks a function provided by N-terminal HEAT repeats.

### Tor1 N-terminal HEAT repeats were required for growth acceleration in phosphate-replete conditions

During invasion of the host, *C*. *albicans* apparently experiences starvation for inorganic phosphate, since expression of the high-affinity inorganic phosphate transporters Pho84 and Pho89 is induced in models of invasive disease [49–52], and since loss of Pho84 attenuates virulence [53]. We examined the role of Tor1 and its N-terminal HEAT repeats in distinct conditions of phosphate availability. *TOR1* wild type, heterozygotes (*tor1/TOR1*), Del381 and FL cells were grown in different phosphate concentrations. In low ambient phosphate, cells overexpressing *TOR1-Del381* in the absence of doxycycline grew at rates comparable to those overexpressing *TOR1-FL* (Fig 2C); they showed mildly increasing growth defects with increases in phosphate concentrations (Fig 2C). During partial repression of *tetO*, Del381 cells showed increasing relative growth defects in increasing ambient phosphate concentrations compared with cells containing full length *TOR1* alleles (Fig 2C). We concluded that the N-terminal HEAT repeats were required for growth acceleration in favorable phosphate- as well as nitrogen source conditions.

### Tor1 N-terminal HEAT repeats contributed to acceleration of growth in high glucose and were required for growth on non-fermentable carbon sources and for physiologic oxygen consumption

**Fig 3:**
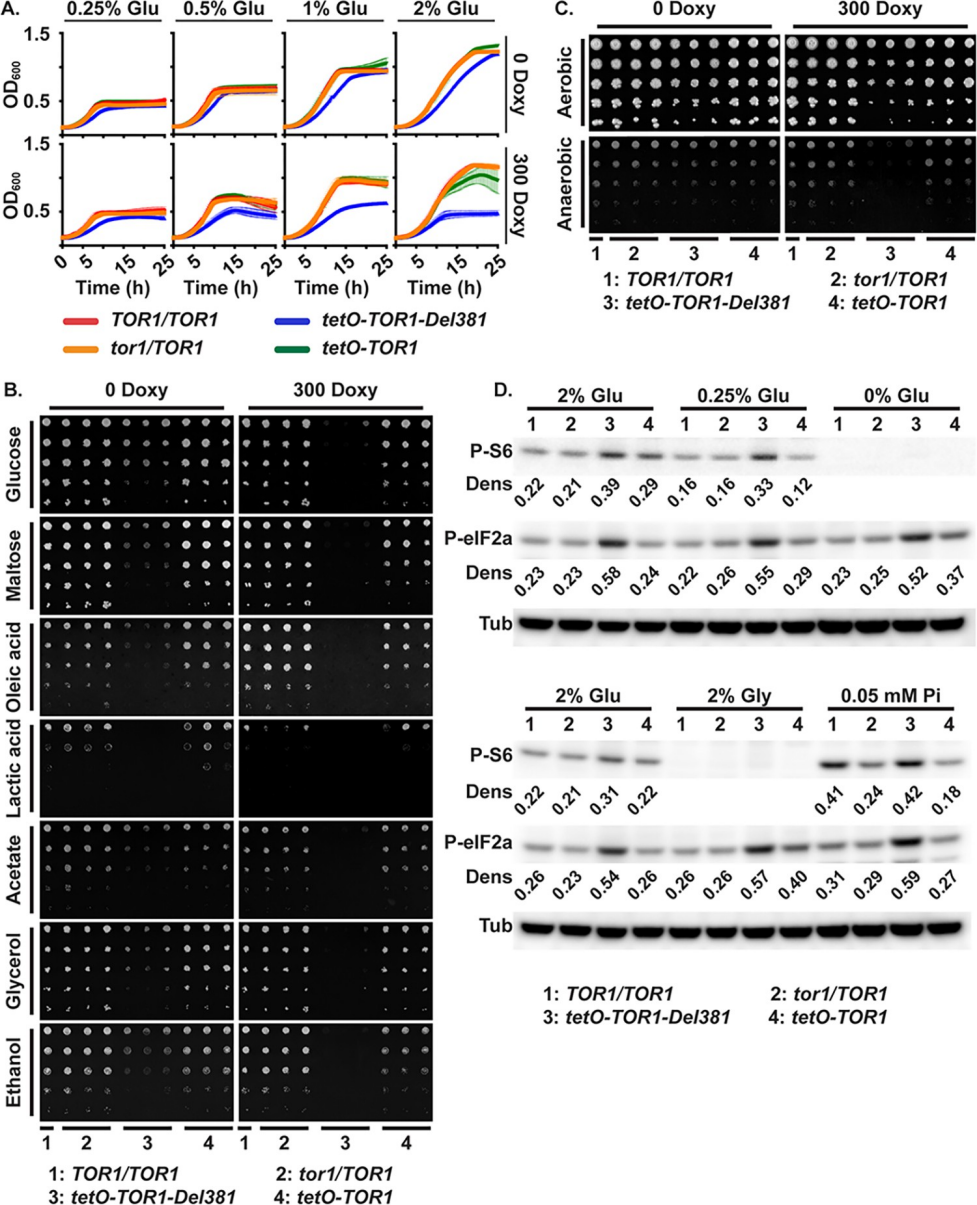
Cells lacking Tor1 N-terminal HEAT repeats failed to adapt growth to carbon source availability and were defective in growth on non-fermentable carbon sources. **A.** Cells were grown in YNB medium with varying concentration of glucose (Glu), without or with 300 ng/ml doxycycline (300 Doxy). **B.** Cells of indicated genotypes were spotted onto YNB without inositol agar medium containing different carbon sources (2% w/v), without or with 300 ng/ml doxycycline. (*TOR1/TOR1*, JKC1713; *tor1/TOR1*, JKC1345, JKC1346, JKC1347; *tetO-TOR1*-*Del381*, JKC1442, JKC1445, JKC1441; *tetO-TOR1*, JKC1543, JKC1546, JKC1549). **C.** Cells of indicated genotypes were spotted onto YPD agar medium without or with 300 ng/ml doxycycline and incubated either aerobically or anaerobically for 48 h. **D.** Western blot. Cells pre-grown in YPD with 5 ng/ml doxycycline for 4 h were inoculated into YNB with 5 ng/ml doxycycline containing final nutrient concentrations as indicated (2% Glu: 2% glucose, 10 mM Pi; 0.25% Glu: 0.25% glucose, 10 mM Pi; 0% Glu: no added direct carbon source, 10 mM Pi; 2% Gly: 2% glycerol, 10 mM Pi; 0.05 mM Pi: 2% glucose, 0.05 mM Pi). Protein extracts were probed with antibody to phosphorylated Rps6 (P-S6) or phosphorylated eukaryotic translation initiation factor 2A (P-eIF2a), and tubulin (Tub) as loading control. Dens: signal intensity ratio of P-S6 or P-eIF2a to Tub. (*TOR1/TOR1*, JKC1713; *tor1/TOR1*, JKC1346; *tetO-TOR1-Del381*, JKC1445; *tetO-TOR1*, JKC1546). Same Western blotting membranes were sequentially probed with P-S6 antibody and then P-eIF2a antibody.

Carbon source- and phosphate repletion are components of *C*. *albicans* cells’ nutrient status monitored by TORC1 [25,33]. In the host interaction, *Candida* cells compete with host phagocytes for glucose [54–56]. During growth in low concentrations of glucose, cells overexpressing *TOR1-Del381* from *tetO* grew at similar rates as wild type or cells expressing full length *TOR1* (Fig 3A). In high glucose concentrations, Del381 cells grew more slowly than FL cells; this effect was more pronounced during partial repression of *tetO* with 300 ng/ml doxycycline (Fig 3A). This finding suggested that functions residing in the N-terminus of the N-HEAT domain contributed to growth acceleration during repletion of the preferred carbon source, glucose.

*C*. *albicans* does not encounter high glucose concentrations during invasive infection: human bloodstream glucose concentration is 0.1%. *Candida*’*s* ability to use a range of carbon sources contributes to fitness in the host [54, 57–64]. The contribution of Tor1 and its N-terminal HEAT repeats to utilization of another fermentable carbon source, maltose, and of the non-fermentable carbon sources oleic acid, lactate, acetate, glycerol and ethanol were examined. Del381 cells had growth defects on all non-fermentable carbon sources (Fig 3B). On fermentable carbon sources their growth defects during *tetO* induction compared with FL cells were minor (Fig 3B). During partial *tetO* repression, Del381 cells had more severe growth defects than FL cells (Fig 3B). Del381 cells’ growth defect on lactate and acetate compared to wild type was not due to acid stress, because it was not increased on glucose- or glycerol-containing medium buffered to pH 2 (Fig C in S1 Text). Physiologic expression of full-length *TOR1* was required for adequate use of non-fermentable carbon sources.

Since utilization of fermentable carbon sources like glucose and maltose was less impaired in Del381 cells compared with use of non-fermentable carbon sources like oleic acid and ethanol (Fig 3B), we asked whether these cells were able to grow anaerobically, i.e. under conditions requiring fermentation. Del381 cells had no specific growth defect under anaerobic (hypoxic) conditions compared with wild type during overexpression of *TOR1-Del381* in the absence of doxycycline (Fig 3C); only when *TOR1-Del381* transcription was partially repressed were these cells unable to grow anaerobically (Fig 3C). We concluded that fermentation can proceed without a regulatory activity residing in the Tor1 N-terminal HEAT repeats, but that this region of Tor1 may contribute to regulating fermentative glucose metabolism.

TORC1 activation and translational regulation through eIF2α were examined in cells growing in 2% glucose (control), 0.25% glucose, 2% glycerol and without a carbon source. The P-S6 signal, indicating TORC1 activation, was slightly reduced in the lower glucose concentration (Fig 3D). Del381 cells showed aberrantly increased P-S6 intensity in low as well as high glucose concentrations. In the absence of a direct carbon source (0 glucose) and in 2% glycerol, the P-S6 signal was undetectable for all strains. Phosphorylation of eIF2α did not change during provision of different glucose concentrations or glycerol (Fig 3D), but the P-eIF2α signal again was stronger in Del381 cells under these conditions, whether or not they had a detectable P-S6 signal. *C*. *albicans* Gcn2 signaling apparently did not respond to carbon source provision under these experimental conditions; it was uncoupled from TORC1 activation in cells lacking N-terminal HEAT repeats. Coordination of translation with carbon source availability may therefore require Tor1 N-terminal HEAT repeats.

### Respiration defects and distinct responses to inhibition of electron transport chain components in Del381 cells suggest specific Tor1 roles in respiration

**Fig 4:**
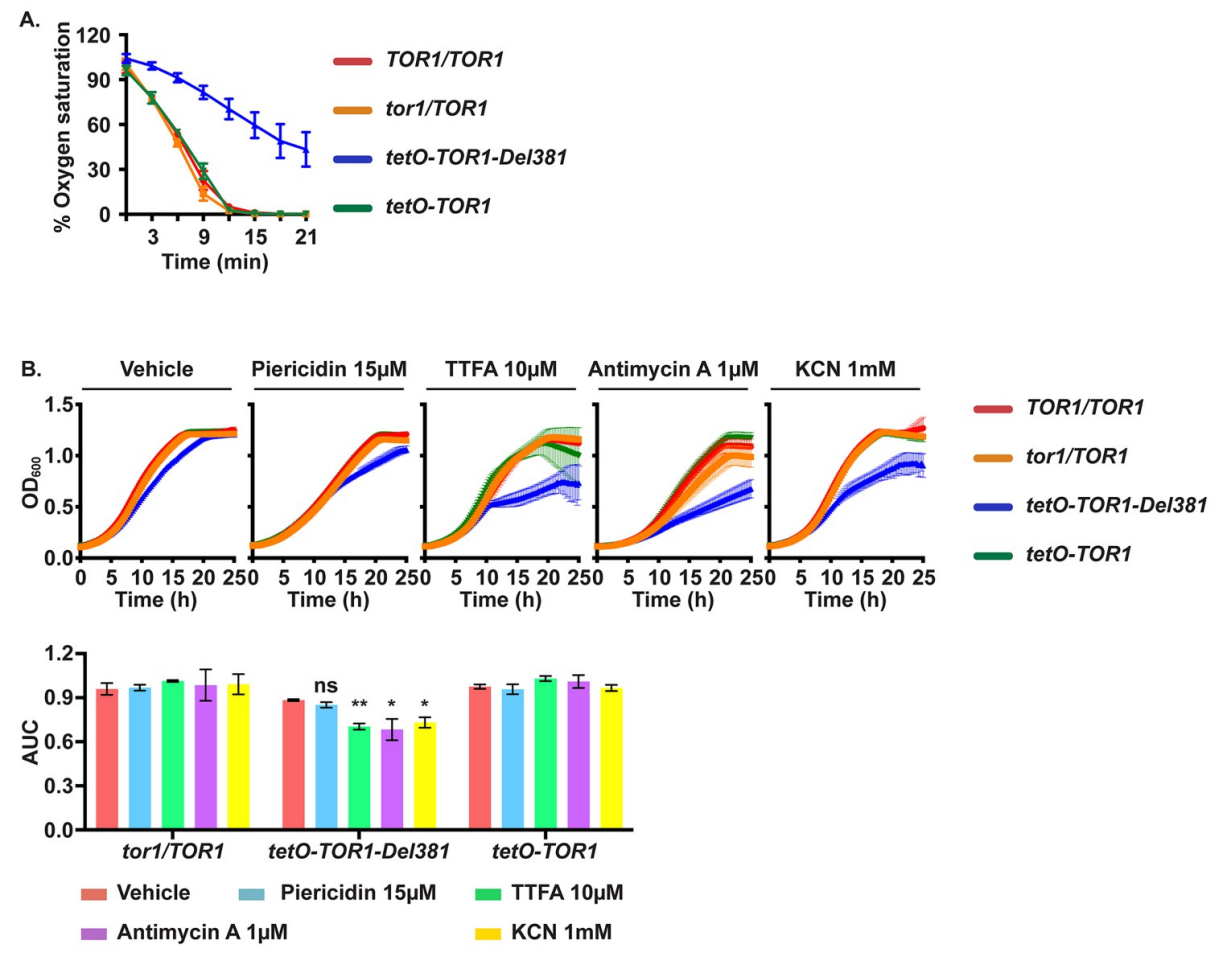
Cells lacking Tor1 N-terminal HEAT repeats were defective in oxygen consumption and hypersensitive to electron transport chain complex inhibitors. **A.** Percentage of oxygen saturation of the medium in strains with distinct *TOR1* alleles grown in YPD. Error bars show SD of 3 biological replicates. (*TOR1/TOR1*, JKC1713; *tor1/TOR1*, JKC1347; *tetO-TOR1-Del381*, JKC1441; *tetO-TOR1*, JKC1549). **B.** Cells of indicated genotypes were pre-grown in YPD and inoculated in YNB with 2% glucose without inositol, containing Vehicle (DMSO), Piericidin, Thenoyltrifluoroacetone (TTFA), Antimycin A or potassium cyanide (KCN) at the indicated concentrations. Error bars show SD of 3 technical replicates. (*TOR1/TOR1*, JKC1713; *tor1/TOR1*, JKC1347; *tetO-TOR1-Del381*, JKC1441; *tetO-TOR1*, JKC1549). The upper panel shows the actual growth curves and the lower panel shows the corresponding area under the curve (AUC) for each condition. ns is *p* = 0.080 (Piericidin); ** is *p* = 0.0026 (TTFA); * is *p* = 0.040 (Antimycin A) and *p* = 0.016 (KCN); error bars show SD of 3 technical replicates. Representative of 3 biological replicates.

We asked whether lagging growth of Del381 cells in high glucose concentrations in aerobic conditions corresponded to decreased respiration. We measured oxygen consumption of wild type, *tor1/TOR1*, Del381 and FL cells. Oxygen consumption was significantly decreased in Del381 cells in the absence of doxycycline, consistent with an inadequate respiratory metabolism (Fig 4A). We concluded that N-terminal HEAT repeats contributed to increasing oxidative phosphorylation when glucose was abundant.

Four protein complexes comprise the electron transport chain, in which energy released in a series of redox reactions is used to create a proton gradient across the inner mitochondrial membrane that in a final step powers ATP synthase. Oxygen is consumed by reduction to water in the last redox reaction of the electron transport chain. Given decreased oxygen consumption of Del381 cells growing in abundant glucose (Fig 4A), we questioned whether specific steps in the electron transport chain were defective in these cells. We exposed wild type, *tor1/TOR1* heterozygotes, Del381 and FL cells growing in glucose to inhibitors of specific electron transport chain complexes. Del381 cells had no specific growth defect in piericidin, a Complex I inhibitor (Fig 4B). In contrast, their growth was significantly impaired during inhibition of Complexes II, III and IV by thenoyltrifluoroacetone (TTFA), antimycin A and KCN, respectively (Fig 4B). Interestingly, in the absence of *tetO* inhibition, cells bearing all other *TOR1* genotypes had no growth defects in these electron transport chain inhibitors (Fig 4B). Inhibition specifically of Complex I therefore conferred no further growth defect on Del381 cells, while inhibition of the 3 other complexes had synthetic effects with loss of N-terminal HEAT repeats. We concluded that this region of Tor1 might have specific regulatory roles in the biosynthesis or activity of Complex I of the electron transport chain.

### Superoxide stress endurance required Tor1 N-terminal HEAT repeats

**Fig 5:**
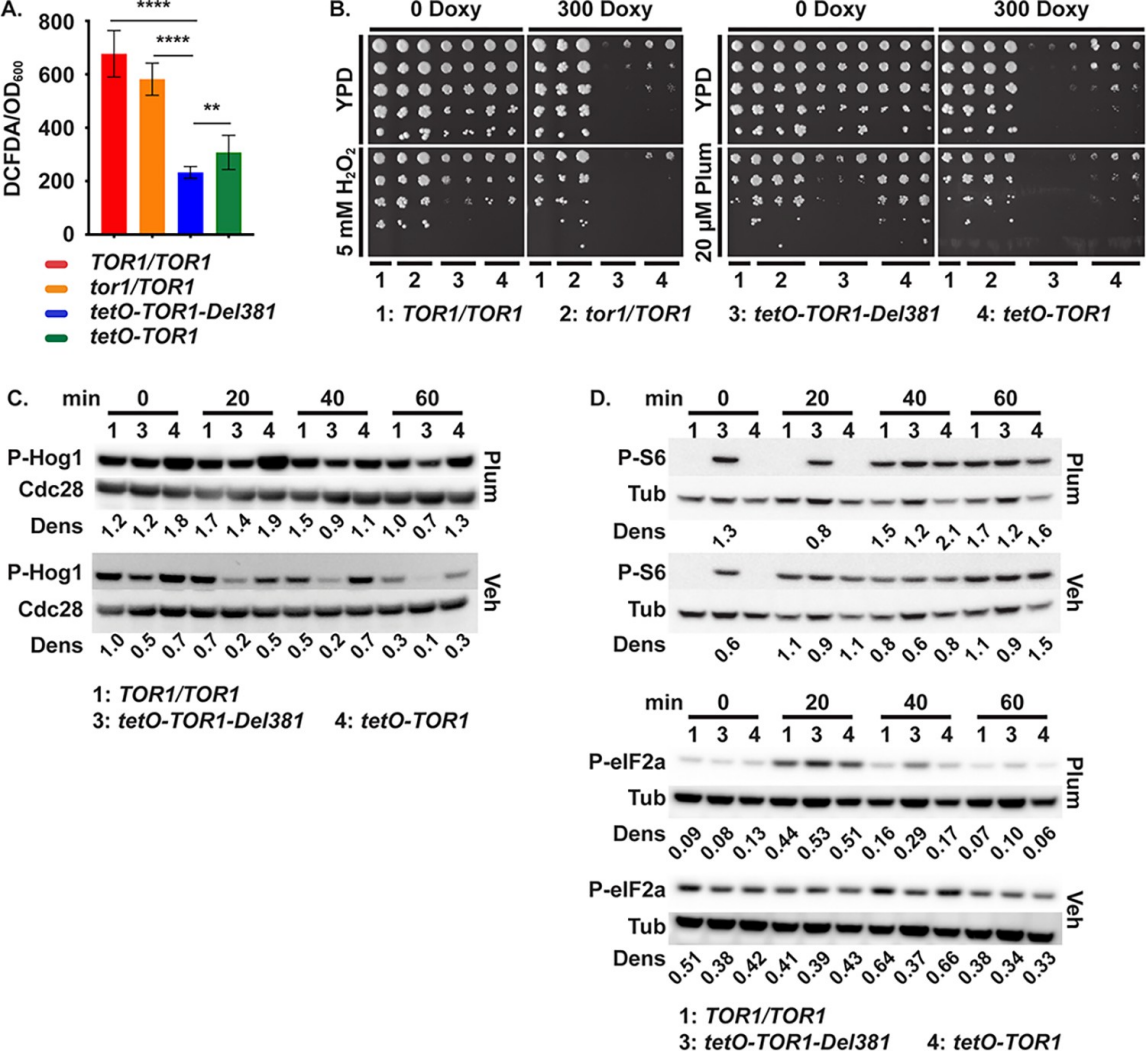
Tor1 N-terminal HEAT repeats were required for oxidative stress responses. **A.** DCFDA-detectable ROS. Cells cultured overnight in YPD were diluted in SC medium (LoFlo) at OD_600_ 0.5 and fluorescence intensity was determined after staining cells with 50 μM DCFDA for 90 minutes. **** is *p*<0.0001; ** is *p* = 0.0079; error bars show SD of 3 biological replicates. (*TOR1/TOR1*, JKC1713; *tor1/TOR1*, JKC1347; *tetO-TOR1-Del381*, JKC1441; *tetO-TOR1*, JKC1549). **B.** Dilutions of cells of indicated genotypes were spotted on YPD medium with or without 300 ng/ml doxycycline (300 Doxy), oxidative stress was induced with 5 mM H_2_O_2_ or 20 μM Plumbagin (Plum). (*TOR1/TOR1*, JKC1361; *tor1/TOR1*, JKC1345, JKC1346, JKC1347; *tetO-TOR1-Del381*, JKC1442, JKC1445, JKC1441; *tetO-TOR1*, JKC1543, JKC1546, JKC1549). **C, D.** Cells of indicated genotypes were pre-grown in YPD medium with 5 ng/ml doxycycline for 3.5 h (Time 0) and then diluted into fresh YPD medium with 5 ng/ml doxycycline with either 10 μM Plumbagin (Plum) or DMSO as vehicle (Veh). Total protein extract was probed with antibody to phosphorylated Hog1 (P-Hog1) and the PSTAIRE antigen of Cdc28 as loading control (**C**), or with antibody to phosphorylated Rps6 (P-S6) and eIF2a (P-eIF2a), and tubulin (Tub) as loading control (**D**). Dens: signal intensity ratio of P-Hog1 to Cdc28 (**C**) or P-S6 or P-eIF2a to Tub (**D**) (*TOR1/TOR1*, JKC1713 for **C** and JKC1361 for **D**; *tetO-TOR1-Del381*, JKC1441; *tetO-TOR1*, JKC1549). Same samples were run on separate gels to detect either P-S6 or P-eIF2a in panel D.

Reduced oxygen consumption of Del381 cells implies a lower rate of mitochondrial activity that generates reactive oxygen species (ROS). DCFDA-detectable ROS were in fact lower in cells with *tetO*-controlled *TOR1* alleles (Fig 5A). We questioned whether decreased intracellular ROS concentrations of FL and Del381 cells might enable better endurance of oxidative stress; alternatively, perturbation of Tor1 could impair the switch from growth-promoting to stress-enduring processes and increase sensitivity to oxidative stress. We examined the role of Tor1 and its N-terminal HEAT repeats in the fungus’s endurance of oxidative stress by exposing cells to the superoxide-generating compound plumbagin and the peroxide H_2_O_2_. *tor1/TOR1* heterozygous cells, spotted in 5-fold dilutions on YPD plates containing plumbagin or H_2_O_2_, were able to tolerate these compounds as well as wild type, but cells expressing *tetO-TOR1-FL* were hypersensitive to H_2_O_2_ in the absence and presence of partial *tetO* repression with doxycycline (Fig 5B). Del381 cells were strikingly hypersensitive to plumbagin even more than to H_2_O_2_ (Fig 5B). These findings confirmed that TORC1 contributes to managing oxidative stress in *C*. *albicans*, and suggested that the N-terminal HEAT repeats of the Tor1 protein were critical for its role in superoxide stress endurance.

Oxidative stress activates signaling through the HOG MAP kinase pathway in *C*. *albicans* as assayed by the Hog1 phosphorylation state [65–67]. This pathway induces antioxidant mechanisms like catalase, superoxide dismutase and enzymes involved in the thioredoxin and glutaredoxin systems [67]. Del381 cells were defective in Hog1 phosphorylation at baseline as well as in response to plumbagin exposure, under conditions in which FL cells induced a strong phospho-Hog1 signal (Fig 5C). This result suggested that Tor1 N-terminal HEAT repeats were required to induce a physiologic Hog1 oxidative stress response.

We tested whether during oxidative stress exposure, TORC1 signaling was physiologically downmodulated in these *tor1* mutants. Wild type and FL cells responded to plumbagin exposure as expected, by inhibiting Rps6 phosphorylation as evinced by an absent P-S6 signal on Western blot in the first 20 minutes of the time course. In contrast, Del381 cells failed to downmodulate P-S6, remaining in an abnormally activated TORC1 state even shortly after exposure to plumbagin (Fig 5D). To exclude an effect of plumbagin on transcription from *tetO*, we examined *TOR1* mRNA levels by qRT-PCR in each of the strains and found no specific effect (Fig D in S1 Text).

Since in *S*. *pombe*, translation initiation during oxidative stress is suppressed by Gcn2 kinase’s phosphorylation of eIF2α, we examined this response in *C*. *albicans* cells from the same cell lysates exposed to plumbagin or vehicle that were assayed for P-S6. At the earliest time point (20 minutes) in vehicle, the P-S6 and P-eIF2α signals of Del381 cells were not higher than in wild type or FL cells (Fig 5D). In contrast, in plumbagin-treated cells after the first 20-minute time point, the P-eIF2α signal of Del381 cells was stronger than in wild type or FL cells (Fig 5D), indicating stronger pro-inhibitory activity by Gcn2. Hence in the absence of stress, Del381 cells’ TORC1- and Gcn2 signaling were aligned. During oxidative stress with plumbagin TORC1- and Gcn2 signaling were uncoupled in these cells, with inappropriately increased TORC1 signaling and increased counter-regulation by inhibitory Gcn2 signaling.

### Downregulation of translation during oxidative stress required Tor1 N-terminal HEAT repeats

**Fig 6:**
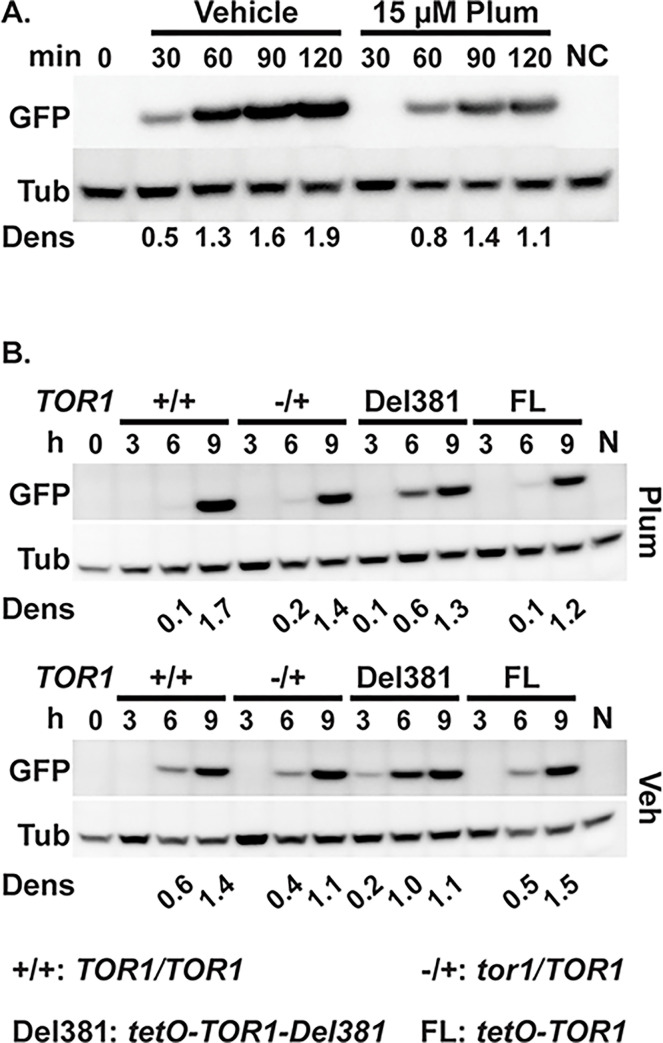
Tor1 N-terminal HEAT repeats were required for oxidative stress-induced delay of translation initiation. **A.** Cells expressing GFP from *tetO* (ON) (strain TETG25B) were grown in YPD medium for 15 h, then inoculated into fresh YPD and pre-grown for 3.5 h (Time 0, 0 min). GFP expression was induced with 50 μg/ml doxycycline in SC medium with Vehicle (DMSO) or 15 μM Plumbagin (Plum). Negative control (NC) cells were grown in SC medium without doxycycline (with DMSO) for 120 min. Total protein extracts were probed with antibody to GFP, and tubulin (Tub) as loading control. Dens: signal intensity ratio of GFP to Tub. Representative of 2 biological replicates. **B.** Cells expressing *pMAL2*-GFP in backgrounds with distinct *TOR1* alleles were pre-grown in YPD medium with 10 ng/ml doxycycline for 15 h (+/+, JKC2616; -/+, JKC2620; Del381, JKC2624; FL, JKC2628). GFP expression was induced by inoculation into YP-Maltose medium with 5 ng/ml doxycycline, containing 13 μM Plumbagin (Plum) or DMSO as vehicle (Veh). (0 h, JKC2616 after pre-growth; N, JKC2616 inoculated into YPD instead of YP-Maltose with 5 ng/ml doxycycline, Plumbagin or DMSO, grown for 9 h). Total protein extracts were probed with antibody to GFP, and tubulin (Tub) as loading control. Dens: signal intensity ratio of GFP to Tub.

Translation initiation of most messages [29] is induced during TORC1 activation and repressed during TORC1 inhibition, with specific regulatory mechanisms known for mTOR [68]. During oxidative stress, as during TORC1 inhibition by rapamycin, translation in *S*. *cerevisiae* is also inhibited through Gcn2-dependent phosphorylation of eIF2α, as well as through decreased ribosomal transit [69]; Gcn2 also responds to oxidative stress to inhibit translation initiation in *S*. *pombe* [70]. Since TORC1 signaling as reflected in the P-S6 signal was hyperactive in Del381 cells during oxidative stress, while paradoxically, their translation inhibition through Gcn2 phosphorylation of eIF2α was also increased, we questioned which signal determined the final output, translational activity. We used a heterologous message, GFP, whose transcription we could control from *tetO* and which presumably had no internal sequences directing translational regulation. We examined whether appearance of the protein was decreased in *C*. *albicans* during exposure to the superoxide generating compound plumbagin. As expected, wild type cells expressing GFP from inducible *tetO* showed slower appearance of a GFP signal during exposure to plumbagin than to vehicle (Fig 6A).

The effect of plumbagin on GFP translation in *tor1* cells in which GFP was expressed from the conditional *MAL2* promoter (*pMAL2*) was then examined. When cells are shifted from glucose to maltose, the *MAL2* promoter is induced; we assayed appearance of a GFP protein signal after this shift in cells containing *TOR1* genotypes wild type, *tor1/TOR1*, Del381 and FL. Translation of GFP to detectable levels occurred earlier in plumbagin- and vehicle-exposed Del381 cells than in FL cells (Fig 6B). Quantitative RT-PCR showed that while plumbagin exposure reduced GFP transcription in cells of all 3 genotypes examined (Fig E in S1 Text), transcriptional differences between these genotypes did not explain the differences in GFP protein levels (Fig E in S1 Text). Together, these findings indicated that translation was aberrantly upregulated in Del381 cells and that the hyperactive TORC1 signal, not simultaneously increased eIF2α phosphorylation and hence inhibition by Gcn2, determined this final output.

### Tor1 N-terminal HEAT repeats participated in the response to cell wall- and heat stress

**Fig 7:**
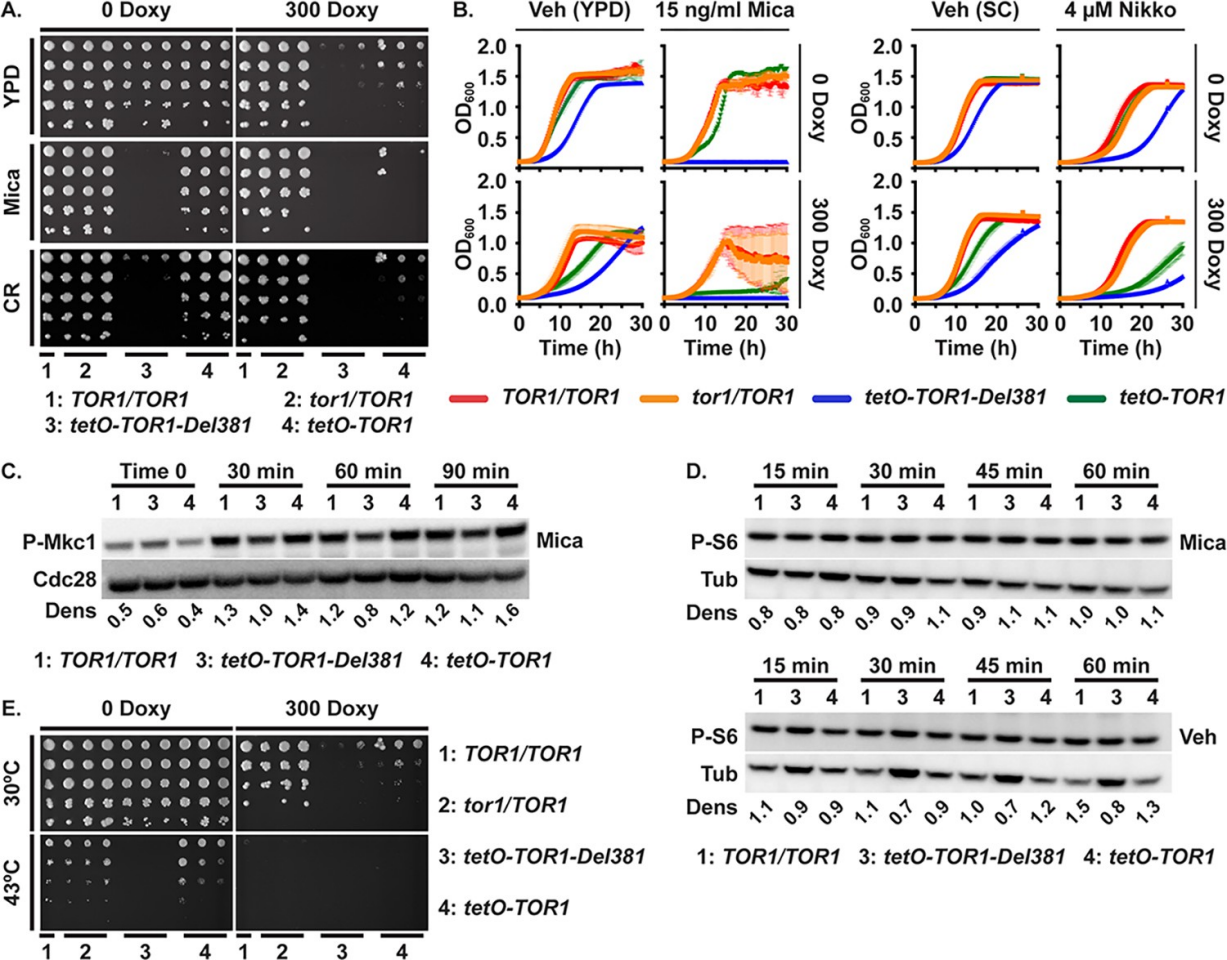
Tor1 N-terminal HEAT repeats were required for adequate cell wall- and heat stress responses. **A, E.** Dilutions of cells of indicated genotypes were spotted on YPD medium without or with 300 ng/ml doxycycline (300 Doxy), cell wall stress was induced with 10 ng/ml micafungin (Mica) or 15 μg/ml Congo red (CR) (**A**), and heat stress was induced at 43^o^ (**E**); strains *TOR1/TOR1*, JKC1361 for YPD and micafungin and JKC1713 for Congo red and heat stress; *tor1/TOR1*, JKC1345, JKC1346, JKC1347; *tetO-TOR1-Del381*, JKC1442, JKC1445, JKC1441; *tetO-TOR1*, JKC1543, JKC1546, JKC1549). **B.** Cells of indicated genotypes were grown in YPD medium containing Vehicle (H_2_O) or 15 ng/ml micafungin (Mica), without or with 300 ng/ml doxycycline (left panel); or in synthetic complete medium (SC) containing Vehicle (H_2_O) or 4 μM nikkomycin (Nikko), without or with 300 ng/ml doxycycline (right panel). Strains *TOR1/TOR1*, JKC1713; *tor1/TOR1*, JKC1347; *tetO-TOR1-Del381*, JKC1441; *tetO-TOR1*, JKC1549. Error bars show SD of 3 technical replicates. **C, D.** Cells of indicated genotypes were pre-grown in YPD medium with 5 ng/ml doxycycline for 3.5 h (Time 0) and then diluted into fresh YPD medium with 5 ng/ml doxycycline with either 10 ng/ml micafungin (Mica) or H_2_O as vehicle (Veh, **D** lower panel is the same P-S6 blot as Fig 1B). Total protein extract was probed with antibody to phosphorylated Mkc1 (P-Mkc1) and the PSTAIRE antigen of Cdc28 as loading control (**C**), or with antibody to phosphorylated Rps6 (P-S6) and tubulin (Tub) as loading control (**D**). Dens: signal intensity ratio of P-Mkc1 to Cdc28 (**C**) or P-S6 to Tub (**D**); strains *TOR1/TOR1*, JKC1713; *tetO-TOR1-Del381*, JKC1441; *tetO-TOR*, JKC1549. Representative of 2 biological replicates.

Host phagocytes exert physical force and break *C*. *albicans* hyphal cells [71]. Mechanical stress and weakening of cell walls induces protein kinase C (PKC)-dependent cell wall integrity responses to prevent *C*. *albicans* cells from rupturing or breaking [72–75]. Activation of these responses can be assayed by the Mkc1 phosphorylation state [74]. In *S*. *cerevisiae*, an upstream cell wall integrity pathway component Rho1, in its GTP-loaded state, displaces the major TORC1 effector Tap42-2A phosphatase that promotes stress responses from its binding site on Kog1, and thereby activates it [76]. In a modulatory loop, Tor kinase function is required for *S*. *cerevisiae* Rho1 activity, while inhibition of TORC1 by rapamycin induces cell wall integrity signaling [76]. We examined the ability of cells lacking Tor1 N-terminal HEAT repeats to respond to cell wall stress. Low concentrations of the beta-1,3-glucan synthase inhibitor micafungin, which induces cell wall stress, were strongly inhibitory to these cells on agar medium even when *tetO-TOR1-Del381* expression was induced in the absence of doxycycline (Fig 7A). The cell wall disrupting dye Congo red, which binds to chitin fibrils [77], similarly had a very strong inhibitory effect on Del381 cells on agar media, regardless of induction or partial inhibition of their *tetO-TOR1-Del381* allele (Fig 7A). Reexamining this phenotype in liquid media, given the activation of cell wall integrity pathway signaling by cells’ contact with agar surfaces [74], we found that *TOR1-Del381-*expressing cells were extremely sensitive to micafungin, whether the allele was overexpressed in 0 doxycycline or partially repressed in 300 ng/ml doxycycline; *TOR1-FL-*expressing cells were also hypersensitive during *tetO* repression but to a somewhat lesser extent (Fig 7B). To confirm that this strong phenotype was not specific to the drug or the cell wall component inhibited (beta-1,3-glucan), we exposed the cells to the chitin synthase inhibitor nikkomycin. Since nikkomycin competes with components of YPD for plasma membrane uptake through oligopeptide transporters, we used synthetic complete medium (SC) for these experiments. Del381 and, to a lesser extent, FL cells were also hypersensitive to nikkomycin during partial *tetO* repression, though the difference to wild type cells’ inhibition was less stark (Fig 7B). The Tor1 N-terminal HEAT domain hence had a distinct role in endurance of cell wall stress.

Cell wall integrity signaling can be assayed in *C*. *albicans* by the Mkc1 phosphorylation state [74]. The P-Mkc1 signal intensity was slightly weaker in Del381 than in FL cells during micafungin exposure, though not at baseline (Fig 7C and 7F in S1 Text), suggesting that the strong growth defect of these cells during cell wall stress was not related to a specific role of the Tor1 N-terminal HEAT region in activating this pathway. Micafungin exposure did not affect Rps-6 phosphorylation even in wild type cells (Fig 7D), confirming our previous observation that TORC1 downmodulation is not part of the physiologic response to cell wall stress [25]. Del381 cells’ hypersensitivity to cell wall stress therefore was not directly related to defective cell wall integrity responses.

Heat stress is encountered by *C*. *albicans* during invasive disease as a component of the host immune response [78]. In *S*. *cerevisiae*, GTP-loaded Rho1 promotes not only cell wall, but also heat stress resistance by displacing Tap42-2A phosphatase from Kog1 [76]. To test whether Tor1 N-terminal HEAT repeats participate in the response to heat stress, we grew cells at 43°C in the absence and presence of sorbitol osmotic rescue which can separate direct heat stress from cell wall stress caused by heat. Growth defects at 43^o^, most severe in Del381 cells, were not osmotically rescued by 1 M sorbitol in any genotype (Fig F in S1 Text), indicating heat stress hypersensitivity in cells with dysregulated Tor1. Del381 cells failed to grow at this elevated temperature, regardless whether *tetO* was induced or repressed (Fig 7E). Hence a signal conveyed through Tor1 N-terminal HEAT repeats was required in the heat stress response.

During a 60-minute exposure to 41^o^, neither wild type nor *tor1* mutant cells downregulated Rps6 phosphorylation (Fig F in S1 Text), indicating that this translation-inhibitory function is not part of the heat stress response. This finding also suggests that it was not lack of translation inhibition that left Del381 cells unable to tolerate elevated temperatures at which cells expressing a wild type *TOR1* allele could grow.

### Plasma membrane stress induced translation inhibition but Tor1 HEAT repeats were not required for membrane stress endurance

**Fig 8:**
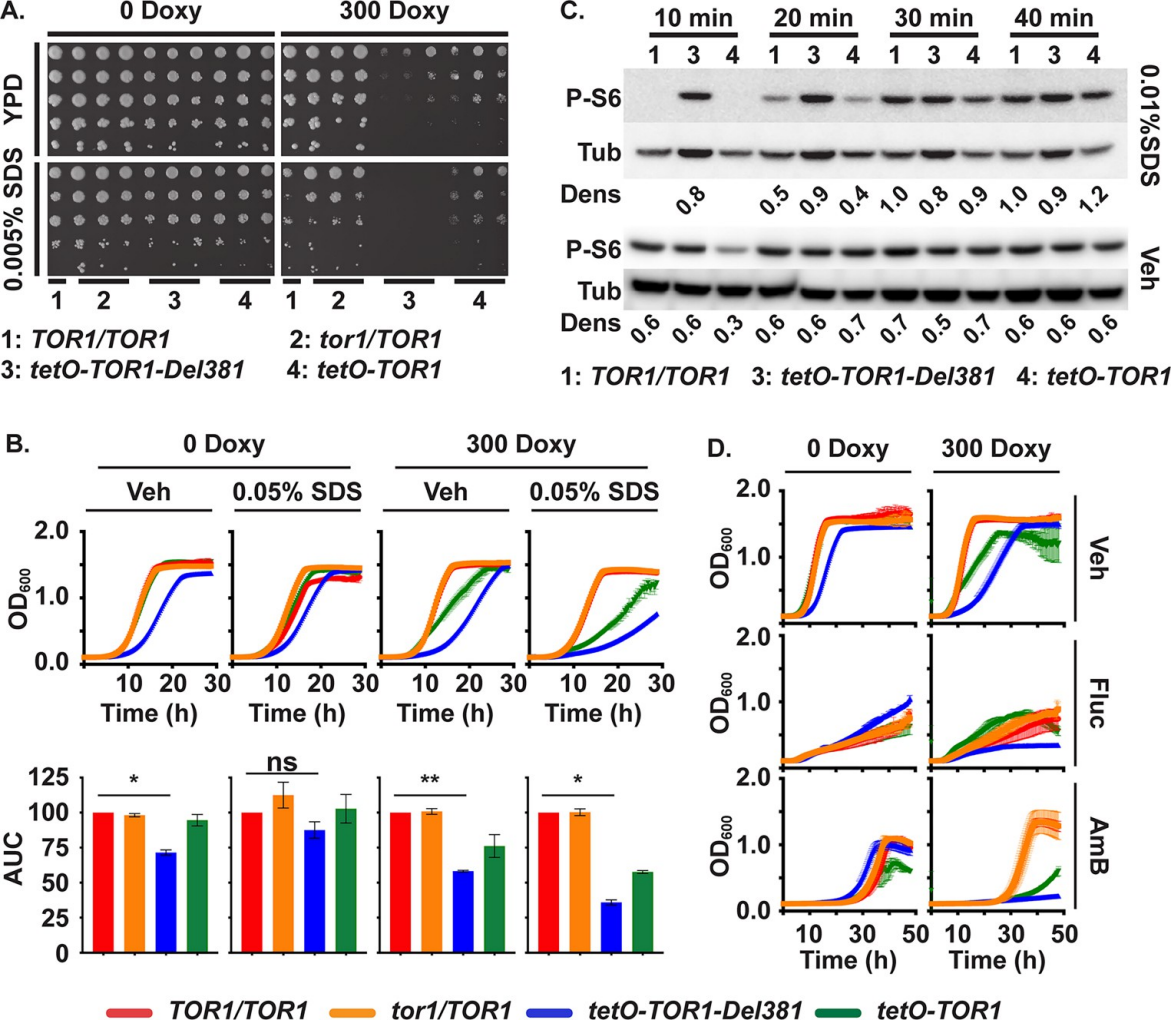
Plasma membrane stress responses did not significantly depend on Tor1 N-terminal HEAT repeats. **A.** Dilutions of cells of indicated genotypes were spotted on YPD medium without or with doxycycline (300 Doxy) and plasma membrane stress was induced with 0.005% SDS. (*TOR1/TOR1*, JKC1713; *tor1/TOR1*, JKC1345, JKC1346, JKC1347; *tetO-TOR1-Del381*, JKC1442, JKC1445, JKC1441; *tetO-TOR1*, JKC1543, JKC1546, JKC1549). **B.** Cells of indicated genotypes were grown in YPD medium containing Vehicle (Veh, H_2_O) or 0.05% SDS, without or with 300 ng/ml doxycycline. Upper panel shows the actual growth curves and lower panel shows the corresponding area under the curve (AUC) for each strain. ** is *p* = 0.0076; * is *p* = 0.029 (Veh, 0 Doxy) and *p* = 0.0129 (0.05% SDS, 300 Doxy); ns is *p* = 0.2, error bars show SD of 2 biological replicates (*TOR1/TOR1*, JKC1713; *tor1/TOR1*, JKC1347; *tetO-TOR1-Del381*, JKC1441; *tetO-TOR1*, JKC1549). **C.** Cells of indicated genotypes were pre-grown in YPD medium with 5 ng/ml doxycycline for 3.5 h, then diluted into fresh YPD medium containing either Vehicle (Veh, H_2_O) or 0.01% SDS and incubated for the indicated times. Total protein extract was probed with antibody to phosphorylated Rps6 (P-S6) and tubulin (Tub) as loading control. Dens: signal intensity ratio of P-S6 to Tub; strains as in panel B. **D.** Cells were grown as in B but treated with 1 μg/ml Fluconazole (Fluc) or 0.2 μg/ml Amphotericin B (AmB).

*C*. *albicans* is exposed to cytoplasmic membrane stress in the host e.g. by bile [79, 80] and by antimicrobial peptides [81]. We used low concentrations of SDS (0.005–0.01%) to test whether Tor1 and its N-terminal HEAT repeats are required in the response to membrane stress. During induction of *tetO-TOR1-Del381* in the absence of doxycycline, Del381 cells grew apparently normally on agar medium containing 0.005% SDS; only during partial repression of *tetO* by doxycycline did residual low-level *TOR1-Del381* expression fail to support growth on this medium (Fig 8A).

Even more striking than on solid media, cells overexpressing *tetO-TOR1-Del381* had a smaller growth defect, compared with wild type, in liquid SDS-containing medium than in vehicle (Fig 8B), i.e. their relative growth defect was partially rescued by SDS exposure. Membrane stress exposure by 0.01% SDS in liquid medium delayed Rps6 phosphorylation in wild type and FL cells while Del381 cells were defective in this response (Fig 8C). Repression of translation hence appeared to be a non-critical component of the physiologic response to plasma membrane stress, since Del381 cells’ defect in this response did not correspond to a growth defect.

Antifungal drugs like fluconazole and amphotericin B perturb plasma membrane function by inhibiting ergosterol biosynthesis or binding ergosterol, respectively. Cells overexpressing *tetO-TOR1-Del381* in the absence of doxycycline were not hypersensitive to these agents (Fig 8D); compared to wild type cells their growth defect may, if anything, have been less pronounced during exposure to fluconazole and amphotericin B than during growth in vehicle (Fig 8D), reminiscent of their growth in SDS. Taken together, these findings indicate that the role of N-terminal HEAT repeats in plasma membrane stress endurance was minor and separable from their role in controlling translation.

### Perturbation of Tor1 expression and loss of its N-terminal HEAT repeats led to hyphal growth dysregulation with distinct phenotypes dependent on environmental conditions

**Fig 9:**
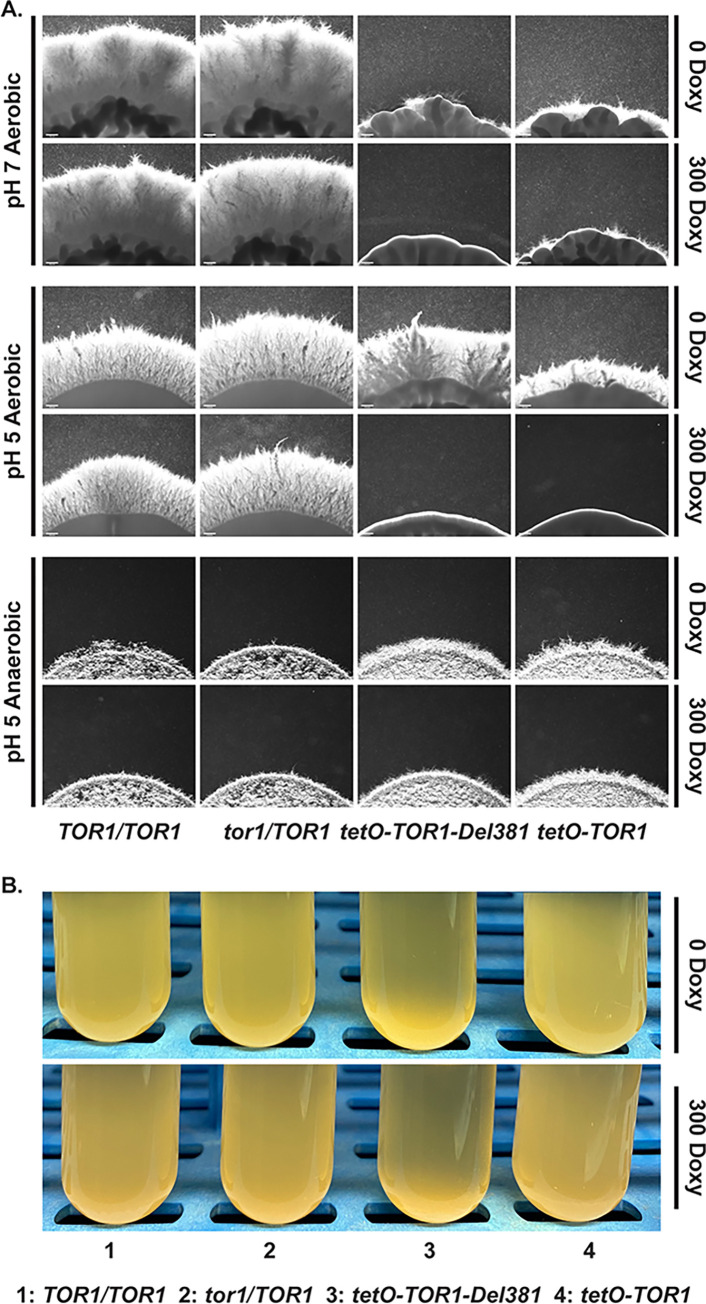
Filamentation responses to distinct stimuli depended on Tor1 N-terminal HEAT repeats. **A.** Cells of indicated genotypes were spotted at equidistant points around agar media, M199 pH 7 165 mM MOPS; M199 pH 5 100 mM MES, and incubated at 37° in aerobic or anaerobic conditions, without and with 300 ng/ml Doxycycline (300 Doxy). Spot edges were imaged. Scale bar 200 μm. **B.** Cells of indicated genotypes were pre-grown in YPD medium with 100 ng/ml doxycycline until exponential phase for 4 h. Flocculation was induced in Spider medium without or with 300 ng/ml Doxycycline (*TOR1/TOR1*, JKC1713; *tor1/TOR1*, JKC1347; *tetO-TOR1-Del381*, JKC1441; *tetO-TOR1*, JKC1549).

Hyphal growth is a result of multiple signaling pathways [82–84]. TORC1 signaling is known to be critical in *C*. *albicans* morphogenesis [37–40]. We examined hyphal growth on several filamentation-inducing media including M199 (Fig 9A), Spider and RPMI (not shown); mutants in *TOR1* invariably had dysregulated filamentation. Del381 and FL cells had decreased hyphal growth during overexpression of their *TOR1* alleles in the absence of doxycycline, and during their partial repression (Fig 9A). During anaerobic growth on acidic medium, cells overexpressing *TOR1-Del381* and *TOR1-FL* from *tetO* were hyperfilamentous, while partial repression of *tetO* resulted in a hyperfilamentous phenotype of FL but not of Del381 cells (Fig 9A). Together these results indicated that the Tor1 N-terminal HEAT repeats, like the entire Tor1 protein, participated in a variety of signaling events whose final output is hyphal growth and which are modulated depending on multiple external and internal cellular parameters.

TORC1 inhibition with rapamycin was previously found to induce aggregation of *C*. *albicans* cells in liquid Spider medium through induction of adhesin gene expression [38]. We questioned whether Tor1 N-terminal HEAT repeats play a role in aggregation. Del381 cells aggregated excessively even during derepression of *tetO*-*TOR1-Del381* in the absence of doxycycline (Fig 9B); aggregation was somewhat more pronounced during partial *tetO* inhibition with 300 ng/ml doxycycline (Fig 9B). Hence Tor1 N-terminal HEAT repeats contribute to repressing aggregation.

Colony surface wrinkles are a filamentation phenotype characterized by Homann et al. for transcriptional regulator mutants [85]. On YPD+10% serum agar medium and on Spider medium, cells containing each of the *TOR1* alleles, in strains from different heterozygous lineages, had reproducible surface wrinkling phenotypes, each of which was distinctive for the mutant allele and for the agar medium (Fig G in S1 Text). We concluded that Tor1 N-terminal HEAT repeats had an important role in signaling events that control filamentation phenotypes.

### Transcriptional analysis of FL cells showed regulation of classic TORC1-controlled genes

**Fig 10:**
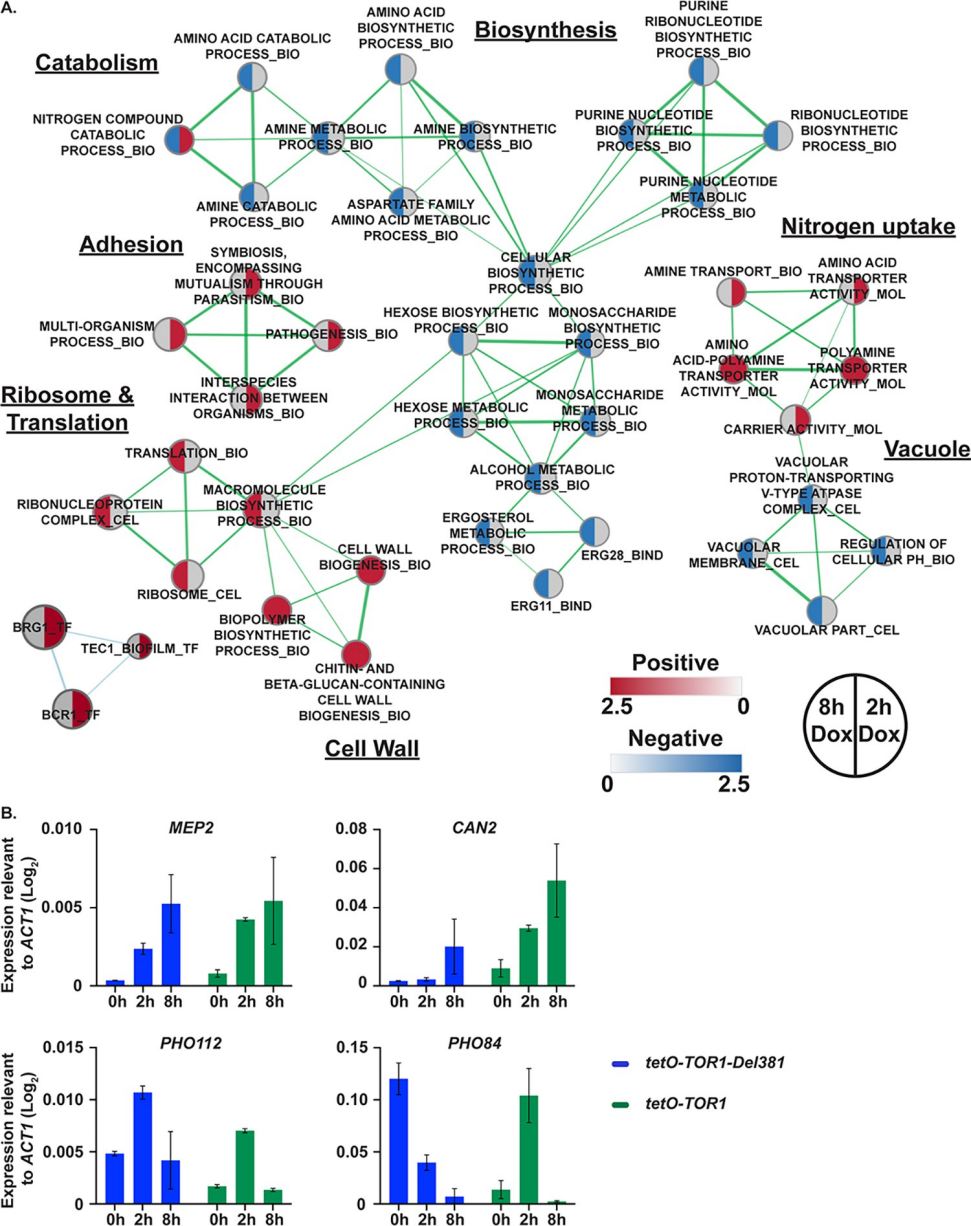
Classic TORC1-regulated gene sets were dysregulated during *TOR1* repression in FL cells. **A.** Enrichment map showing the major categories of genes identified by Gene Set Enrichment Analysis (GSEA) in the transcript profiles of *tor1/tetO-TOR1* (JKC1549) cells repressed in 30 mg/ml doxycycline for 2 h or 8 h in YPD compared to *tor1*/*TOR1* (JKC1347). Nodes represent gene sets (FDR q <0.1) where color intensity reflects the Normalized Enrichment Score (NES) and node size reflects the number of enriched genes. Grey color indicates category was not significantly enriched under this experimental condition. Width of connecting lines (edges) indicates the degree of overlap between categories. **B.** *MEP2*, *CAN2*, *PHO112* and *PHO84* gene expression quantification relative to *ACT1* expression, after repressive (30 mg/ml) 2h and 8h doxycycline treatment in *tor1/tetO-TOR1-Del381* (*tetO-TOR1-Del381*, JKC1441) and *tor1/tetO-TOR1* (*tetO-TOR1*, JKC1549) strains.

In order to identify the set of genes regulated by *TOR1* in *C*. *albicans*, we carried out whole genome transcript profiling of FL cells with fully repressed *tetO* through exposure to high doxycycline concentrations (30 μg/ml) for 2 and 8 h in YPD and compared them to *tor1/TOR1* heterozygotes grown under the same conditions. Depletion of *TOR1* for 2 h resulted in differential expression (≥2-fold, p<0.05) of 520 genes (310 up and 210 down). To identify the categories of cellular functions affected by *TOR1* depletion, we carried out Gene Set Enrichment Analysis (GSEA) as described by Uwamahoro et al. [86] and Sellam et al. [87]. We identified many of the expected Tor1-regulated starvation responses, including downregulation of biosynthetic processes including amino acid and nucleotide biosynthesis (Fig 10A). Concurrent with this we observed increased expression of genes involved in amino acid and nitrogen uptake (including *MEP2* and *CAN2*; Fig 10B), amino acid catabolism, adhesion and biofilm formation (Fig 10A). Comparison of these data with the published data for rapamycin exposure in *C*. *albicans* identified a strong core response significantly enriched for induced genes assigned to the GO terms for basic amino acid transport (*GAP2*, *CAN1*, *CAN2*), transport (*MEP2*, *OPT1*), biofilm formation (*ALS1*, *ALS2*, *YWP1*) and organonitrogen compound catabolism (*PRC2*, *AMO1*, *ARO10*) (S5 File). Genes required in phosphate homeostasis, encoding the predicted secreted phosphatase Pho112 and the inorganic phosphate transporter Pho84 were strongly regulated over the 2 h and 8 h time points (Fig 10B).

At 8 h growth in the presence of doxycycline we observed a large increase in transcription of genes involved in rRNA processing and ribosome biogenesis, possibly as a result of counterregulatory processes. Transcription of genes required in biogenetic processes of glucan- and chitin-containing cell wall components was also upregulated; in light of decreased transcription of carbon source metabolic genes and purine nucleotide biosynthetic genes, we also interpret this finding as a counterregulatory mechanism when cell wall biosynthetic enzymes lacked their nucleotide sugar substrates [88]. Vacuolar components, V-ATPase and pH homeostatic genes were downregulated significantly at 8 h, consistent with control of V-ATPase assembly by *S*. *cerevisiae* TORC1 [89, 90] and with the connection between sugar catabolism and pH homeostasis [91,92]. Biofilm-associated genes and pathogenesis-related genes were upregulated at the 2 h time point; significantly increased expression of genes that interact with the transcription factors Bcr1 and Brg1, as well as Tec1, was noted at the 2 h time point and receded by 8 hours (Fig 10A).

Overall, suppressing transcription of *TOR1-FL* from *tetO* revealed that *C*. *albicans* TORC1 loss of function affects multiple interlocking processes related to transitions between anabolism and catabolism, responses to favorable- versus stress conditions. Our findings are consistent with and expand upon a previous analysis in which rapamycin was used to induce loss of *C*. *albicans* TORC1 function [38]. Also in line with that work and with the partial loss of function phenotypes induced by partial *tetO-TOR1-FL* repression, adhesion and filamentous growth-associated processes and transcriptional regulators were highlighted by this GSEA.

### Cells lacking N-terminal HEAT repeats showed dysregulation of multiple transcriptional modules comprising metabolism, stress responses and the white cell type

**Fig 11:**
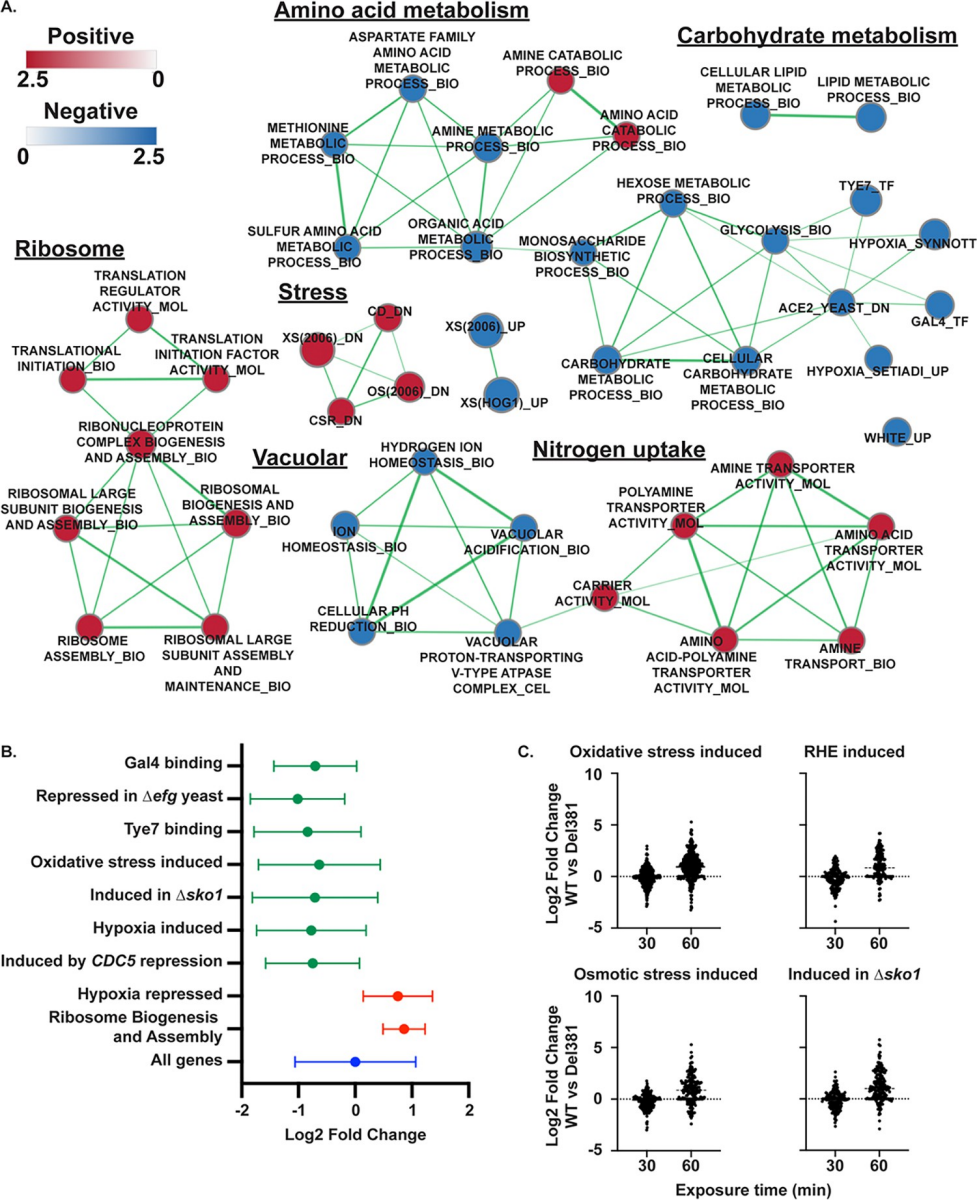
Cells containing derepressed *TOR1-Del381* upregulated ribosome biogenesis and translation related genes but decreased expression of carbon metabolism- and stress response genes. **A.** Enrichment map showing the major categories of genes identified by Gene Set Enrichment Analysis (GSEA) in the transcript profile of *tor1/tetO-TOR1-Del381* cells (JKC1441) grown in YPD without doxycycline compared to the heterozygote *tor1/TOR1* (JKC1347). Nodes represent gene sets (FDR q <0.1) where color intensity reflects the Normalized Enrichment Score (NES) and node size reflects the number of enriched genes. Width of connecting lines (edges) indicates the degree of overlap between categories. **B.** Gene set enrichment analysis was used to identify major categories of genes significantly regulated (adjusted P value < 0.001) in *tor1/tetO-TOR1-Del381* cells (JKC1441) compared to the *tor1/TOR1* control (JKC1347). Graph shows the fold change (Log2) of genes in each indicated category in Del381 cells relative to *tor1/TOR1* cells. Full results in S1 File and S2 File. **C.** Specific gene categories were altered in cells lacking N-terminal HEAT repeats during superoxide stress. Comparison of gene expression in wild type (WT, SC5314) relative to *tor1/tetO-TOR1-Del381* (JKC1441) in the presence of plumbagin (10 μM) after 30 and 60 minutes. Gene categories shown were significantly represented (Q < 0.001) among genes exhibiting increased expression in the wild type relative to *tor1/tetO-TOR1-Del381* cells following 60 minutes incubation in plumbagin. Gene categories are those identified by Enjalbert et al. (oxidative and osmotic stress;[67]), Rauceo et al. *(Δsko1*; [112]) and Spiering et al. (Reconstituted human epithelium induced; [113]).

Microarray analysis was used to examine the transcriptome of Del381 cells compared to heterozygous *tor1/TOR1* cells in the absence of doxycycline repression. Overall, similar processes were regulated as in doxycycline-exposed FL cells, including upregulation of amino acid transporters, downregulation of carbon source metabolism, nucleotide biosynthesis and amino acid metabolism and of vacuolar components and the V-ATPase (Fig 11A). GSEA showed that during *tetO* derepression, Del381 cells decreased expression of genes bound by transcriptional regulators Gal4 and Tye7 [93], and those whose induction requires Efg1 [94] (Fig 11B). Decreased expression of genes associated with stress responses, including oxidative stress [67] and the response to hypoxia [87] was notable (Fig 11A,B, S1 and S2 Files). Indicating a role for TORC1 and specifically N-terminal HEAT repeats in the white-opaque switch, *WH11* was among the most highly upregulated genes in Del381 cells compared with *tor1/TOR1* cells (S1 File).

Del381 cells showed consistent upregulation of gene sets comprising ribosome biogenesis and translation initiation under these conditions of *tetO* derepression, as they also did during *tetO* repression. These findings hint at an anti-anabolic regulatory function residing in the missing N-terminal HEAT repeats of Tor1 whose lack manifests as increased production of translational machinery and increased translation, regardless of the level of transcript that encodes the remaining regions of the protein.

We then examined the capacity of Del381 cells to mount a protective transcriptional response to oxidative stress induced by plumbagin. Exposure of wild type *C*. *albicans* cells to plumbagin induced a transcriptional program indicative of a strong oxidative stress response following 60 min plumbagin exposure relative to cells treated with DMSO alone (S7 File). When the responses of the Del381 and wild type cells were compared, gene categories associated with stress and environmental responses were more strongly induced in the wild type, indicating that N-terminal HEAT repeats of Tor1 were required for effective transcriptional responses to superoxide stress (Fig 11C; S7 and S8 Files) [67, 95]. These results were consistent with Del381 cells’ hypersensitivity to oxidative stress (Fig 5B). In the response to this important host-derived stress, Tor1 N-terminal HEAT repeats were therefore required both at the level of immediate stress signaling and of transcriptional regulation.

In line with increased expression of hypoxia-induced genes [87], dysregulation of genes involved in ergosterol biosynthesis (*ERG1*, *ERG3*, *ERG25*) was observed during repression of *TOR1-Del381* (S3 File). Del381 cells had a 3-fold increase in expression of the fluconazole efflux pump encoding gene *MDR1* (S3 File). These findings might explain these cells’ relative fluconazole resistance.

### Tor1 N-terminal HEAT repeats were required in expression of specific subsets of TORC1-controlled modules including cell wall biosynthetic genes, hyphal transcriptional regulator-controlled genes and mitochondrial genes

**Fig 12:**
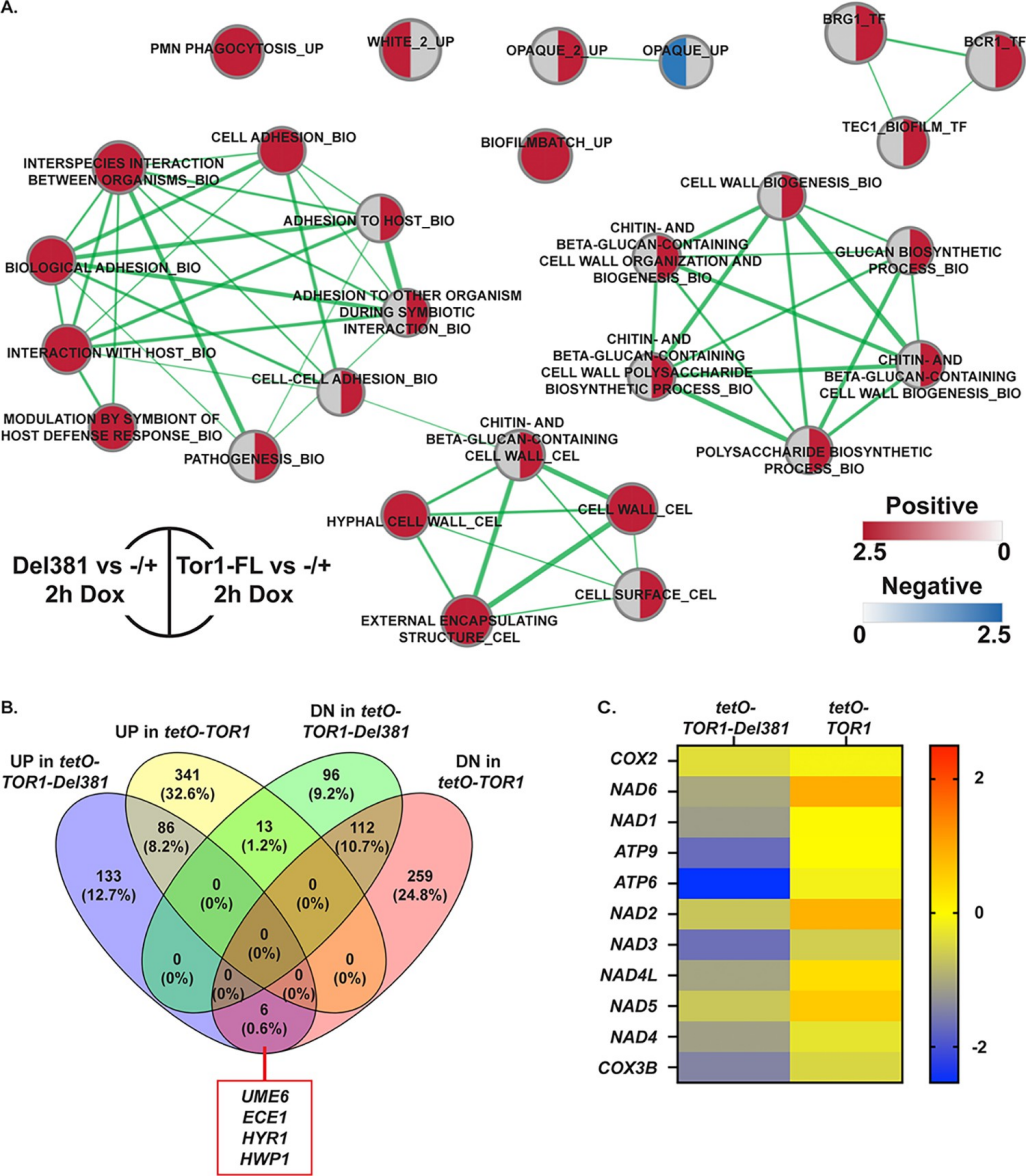
Comparison of gene set expression changes during repression of *TOR1* and *TOR1-Del381* in FL and Del381 cells exposed to doxycycline. **A.** Enrichment map showing the major categories of genes identified by Gene Set Enrichment Analysis (GSEA) in the transcript profiles of *tor1/tetO-TOR1-Del381* cells (JKC1441, left side) and *tor1/tetO-TOR1* cells (JKC1549, right side) compared to heterozygous *tor1/TOR1* cells (-/+, JKC1347) following *tetO* repression in YPD with doxycycline for 2 h. Nodes represent gene sets (FDR q <0.1) where color intensity reflects the Normalized Enrichment Score (NES) and node size reflects the number of enriched genes. Width of connecting lines (edges) indicates the degree of overlap between categories. Grey indicates the category was not significant under the experimental condition. **B.** Comparison of gene expression changes (≥2-fold up and down) in *tor1/tetO-TOR1-Del381* (JKC1441) and *tor1/tetO-TOR1* (JKC1549) cells exposed to doxycycline for 8 h. Six genes induced in *tor1/tetO-TOR1-Del381* cells were suppressed in *tor1/tetO-TOR1* cells, including *UME6*, *ECE1*, *HYR1* and *HWP1*. **C.** Heatmap showing expression of genes that encode Complex I of the mitochondrial electron transport chain in *tor1/tetO-TOR1-Del381* (JKC1441) relative to the heterozygote *tor1/TOR1* (column 1) and in *tor1/tetO-TOR1* (JKC1549) relative to the heterozygote *tor1/TOR1* (column 2). Expression values are Log2 values relative to *tor1/TOR1* (JKC1347).

We compared the effects of full *tetO* repression with 30 μg/ml doxycycline on Del381 and on FL cells (Fig 12A and 12B; S3–S6 Files). In general, repressing the *TOR1-Del381* allele increased the number of differentially regulated genes identified previously. GSEA analysis indicated that the processes affected during *tetO* repression in Del381 cells were quite similar to those perturbed in these cells without doxycycline (S1–S4 Files) and to those affected in FL cells treated with doxycycline (Fig 12A). Among differentially regulated gene categories during doxycycline repression of *TOR1-Del381* versus of *TOR1-FL*, cell wall biosynthetic functions were prominent (Fig 12A). While processes like phosphate homeostasis were disturbed by both FL and Del381 cells’ exposure to doxycycline (Fig 10B), temporal regulation of specific genes, like that encoding the high-affinity phosphate transporter Pho84, was found to be very different in FL and Del381 cells upon closer examination by qRT-PCR (Fig 10B).

In this comparison, a role of Tor1 and specifically, of N-terminal HEAT repeats in control of white- versus opaque cell type-associated genes emerged clearly. Del381 cells exposed to doxycycline expressed opaque cell-associated genes significantly less than FL cells, while their expression of white cell-associated genes was significantly higher. In *S*. *pombe*, sexual differentiation and meiosis are controlled by TORC1 in response to unfavorable conditions of nutrient starvation [96]. In *C*. *albicans*, sexual differentiation takes the form of the white-opaque cell switch and also occurs in response to specific conditions like hypoxia [97]; we find TORC1 control of this developmental process to also pertain in *C*. *albicans*.

Genes controlled by transcriptional regulators required in filamentation and biofilm development, Brg1, Bcr1 and Tec1, also showed significant dependence on activation by intact Tor1, as their expression level differed significantly between Del381 and FL cells exposed to doxycycline (Fig 12A). In fact, among the 6 genes downregulated at 8 hours’ doxycycline repression in FL- and upregulated under the same conditions in Del381 cells, were hyphal-specific genes like *UME6*, *ECE1* and *HWP1* (Fig 12B). Dysregulation of hyphal-specific genes may partially explain Del381 cells’ conditional hyperfilamentous phenotype (Fig 9A).

Phenotypically, Del381 cells were strongly hypersensitive to cell wall stress (Fig 7A,B) with only minor defects in cell wall integrity signaling (Fig 7C). GSEA analysis found that compared with FL cells, transcription of their cell wall biosynthetic genes was decreased during doxycycline repression (Fig 12A). We questioned whether in addition to perturbation of their carbon source metabolism (Fig 11A) and their phenotypic defects in respiration (Fig 3B) and oxidative phosphorylation (Fig 4B), specific transcriptional defects in mitochondrial genes might explain Del381 cells’ hypersensitivity to cell wall stress. Lack of ATP is known to lead to cell wall defects as levels of nucleotide sugar precursors of cell wall polysaccharides drop [88], linking mitochondrial defects with cell wall weakness [35,98–102]. Indeed, Del381 cells had lower expression levels of genes that encode some of the 39 proteins composing Complex I of the *C*. *albicans* electron transport chain (Fig 12C), consistent with hypersensitivity of these cells to inhibitors of electron transport Complexes II, III and IV (Fig 4B) and supporting the connection between intact respiration and cell wall construction observed by other authors [100].

In summary, perturbed levels of *TOR1-Del381* during overexpression or repression from *tetO* expectedly impacted many processes also affected by *TOR1-FL* perturbation: amino acid uptake and -metabolism, translation and the translational machinery, carbon source metabolism, filamentation and adhesion. Specific GSEA details emphasized underappreciated aspects of TORC1 regulation like genes under direct control of the transcriptional regulators Bcr1, Brg1 and Tec1, and white-opaque cell transitions. Alterations specific to Del381 cells observed in the phenotypic analysis were also reflected in the transcription experiments. Cells lacking N-terminal HEAT repeats showed specific transcriptional dysregulation of carbon source metabolism, oxidative phosphorylation, responses to oxidative and osmotic stress, cell wall construction, filamentation and adhesion, exceeding that of FL cells. Transcriptional analysis confirmed that disturbing the function of this least conserved region of the *C*. *albicans* Tor1 kinase disrupts cellular processes known to be required for the interaction with the host.

## Discussion

We set out in this work to examine *C*. *albicans tor1* loss-of-function phenotypes, using genetic perturbation rather than relying on traditional pharmacological inhibition with rapamycin. We then asked whether specific roles could be assigned to a region of Tor kinase that is highly divergent between fungal and human cells, comprising the 8 most N-terminal HEAT repeats. Cells whose only *TOR1* allele was transcribed from repressible *tetO* revealed specific functions to which this region contributes, when phenotypes of cells expressing an N-terminally truncated Tor1 were compared to those expressing the full length protein. Growth of both *tetO*-controlled *TOR1* genotypes correlated with doxycycline concentrations in YPD medium, with 1 μg/ml doxycycline providing nearly complete repression of Del381 cells’ growth, while a moderate doxycycline concentration of 300 ng/ml caused a detectable, minor growth defect in this medium (Fig 1A). For many experiments going forward, we therefore chose 300 ng/ml doxycycline in order to compare phenotypes in cells that had diminished but not ablated *TOR1* expression. By titrating the doxycycline concentration, we were able to modulate partial loss-of-function phenotypes in Tor1. These mutants will be useful reagents for further analysis of *C*. *albicans* TOR function.

Overall, we observed defects in Del381 cells’ adaptation to nutrient quality and quantity, and to many but not all infection-relevant stressors (Table 2). A major input activating TORC1 signaling is availability and quality of nitrogen sources. Lack of N-terminal HEAT repeats had little impact on cells’ growth rates in the non-preferred nitrogen sources proline and tryptophan, in the absence or presence of a moderate repressing doxycycline concentration (Fig 2A). However, in two preferred nitrogen sources, glutamine and ammonium sulfate, Del381 cells grew more slowly in liquid media, and during partial *tetO* repression grew only to low densities (Fig 2A). They similarly failed to maximize growth rates during optimal provision of phosphate- and carbon sources (Figs 2C and 3A). These findings together indicated that the TORC1 response to favorable nutritional conditions, coordinating anabolic processes like translation and DNA replication with provision of metabolic intermediates and maximal harvesting of energy from carbon sources, depends on a function provided by the N-terminal HEAT repeats.

**Table 2:** Del381 cell responses to specific stress types.

Stress type	Hyperintense P-S6	Growth defect	Defective specific pathway signaling
**Non-preferred nitrogen source**	yes	no	not tested
**Low glucose**	yes	no	not tested
**Low inorganic phosphate**	yes	no	not tested
**Oxidative stress**	yes	yes	yes (Hog1 phosphorylation)
**Cell wall stress**	no	yes	no (Mkc1 phosphorylation)
**Heat stress**	no	yes	not tested
**Plasma membrane stress**	yes	no	not tested

Since Del381 cells’ failure to accelerate growth in nutrient-rich conditions occurred in response to three central macronutrients, nitrogen-, carbon- and phosphate sources, we speculate that this defect of N-terminally truncated Tor1 relates not to defective sensing of individual nutrients, but to dysregulation of a pro-anabolic output that normally results from TORC1’s integration of favorable nutritional inputs together with absent perception of unfavorable stressors.

While Del381 cells were unable to appropriately accelerate growth in optimal conditions, they inappropriately activated TORC1 signaling when nutrient conditions were suboptimal, as indicated by an excessive P-S6 signal in the nonpreferred nitrogen source proline and in a low glucose concentration of 0.25% (Figs 2B and 3D). Rps6 phosphorylation was not simply indiscriminately activated, since Del381 cells growing in the absence of glucose (without an added carbon source) or in the nonfermentable carbon source glycerol, had no detectable P-S6 signal, in line with wild type and FL cells (Fig 3D). Hence Del381 cells seemed to be lacking a fine-tuning function of TORC1 activity, while “on” and “off” switches remained functional.

Rps6 phosphorylation, whose biochemical function remains unknown [103], corresponds to translational activation in *C*. *albicans* [25]. If Del381 cells inappropriately activated translation in suboptimal nutrient conditions, their slower growth may have been due to futile protein production with wasteful consumption and premature exhaustion of scarce resources. To examine translational control in these cells by another mechanism, we assayed the behavior of Gcn2 kinase, which inhibits translation, through the phosphorylation state of its target eIF2α. Strikingly, Gcn2 appeared to be hyperactive in Del381 cells in most conditions compared to wild type and FL cells: eIF2α was hyperphosphorylated, indicating inhibition of translation initiation by Gcn2, in conditions where Del381 cells’ P-S6 signal was hyperintense indicating excessive translation activation e.g. during growth in proline (Fig 2B). (Appropriate hyperphosphorylation of eIF2α occurred in Del381 cells in some conditions where the P-S6 signal was undetectable e.g. in stationary phase and in medium without an external carbon source or with glycerol (Figs 2B and 3D)). Experiments we then performed with these cells during oxidative stress may have shed some light on this paradox, as noted below.

Del381 cells were hypersensitive to peroxide- and especially to superoxide stress, as induced by H_2_O_2_ and plumbagin, respectively (Fig 5B). To understand this phenotype mechanistically, we examined their oxidative stress signaling through the Hog1 kinase. Del381 cells’ Hog1 phosphorylation response was weak (Fig 5C), and their transcriptional oxidative stress responses were blunted (Figs 11 and 12A). The *C*. *albicans* HOG pathway has been found by several authors to be linked to TORC1 signaling [39,40,82,104]. Our findings suggest that Tor1 N-terminal HEAT repeats may be involved in the connection between these two signaling modules, and hence required in the host interaction of the fungus.

A rapid response to oxidative stress in the model yeasts *S*. *cerevisiae* and *S*. *pombe* as well as in *C*. *albicans* is widespread inhibition of translation initiation and -elongation, while stress responses are mobilized [69,105–107]; we therefore examined Del381 cells’ Rps6 phosphorylation during superoxide stress (plumbagin exposure). The P-S6 signal of Del381 cells was hyperintense in the first 20 minutes of plumbagin exposure (Fig 5D), indicating inappropriate translation activation. At the same time, translation inhibition through Gcn2 activity was more active than in wild type or FL cells, as assayed by eIF2α phosphorylation (Fig 5D). Since the final common effector of these two signaling pathways is the activity of the translational machinery, we examined which of the two pathways that gave opposing signals—TORC1 an activating- and Gcn2 an inhibitory signal—had the decisive effect, or the “last word.” We examined translation of a heterologous message encoding GFP, with no known intrinsic regulatory motifs responding to oxidative stress, transcribed from regulatable elements, *tetO* or *pMAL2*. In wild type cells, translation of a GFP message transcribed from inducible *tetO* was delayed during oxidative stress exposure (Fig 6A). GFP expressed from *pMAL2* during oxidative stress was translated inappropriately early in maltose-grown Del381 cells compared with wild type, *tor1/TOR1* heterozygous, and FL cells (Fig 6B), indicating that hyperactivation of translation by TORC1 prevailed over translation inhibition by Gcn2.

Why was translation inhibition through Gcn2 upregulated in the same cells (we examined the same protein extract samples for these comparisons of P-S6 and P-eIF2α signal intensities) in which translation was hyperactivated as assayed through P-S6? Since in *S*. *cerevisiae*, Gcn2 directly phosphorylates the N-terminus of Kog1 [108] to downmodulate TORC1 activity, we surmised that the missing HEAT repeats in Del381 cells might participate in downregulation of translation by *C*. *albicans* Gcn2 through Kog1 during oxidative stress; when their activity is absent in these cells, Gcn2 develops compensatory hyperactivity. Excessive GFP translation during oxidative stress in these cells showed that this compensatory upregulation of eIF2α phosphorylation is not sufficient, in the end, to suppress inappropriate translation in Del381 cells.

Hyperactive translation did not invariably predict growth defects however: Del381 cells exposed to the plasma membrane stressor SDS had inappropriately high levels of P-S6 while enduring this stress well when their *TOR1-Del381* allele was not repressed (Fig 8A–8C). Strikingly, during *TOR1-Del381* overexpression, these cells showed no relative growth defect in fluconazole and endured amphotericin B exposure; even during partial *tetO* repression their growth defect in these conditions was minor compared to their defect in vehicle, indicating enhanced fluconazole endurance (Fig 8D). Increased expression of *MDR1* in Del381 cells, which encodes a multidrug efflux pump, might partially explain this phenotype.

Del381 cells were hypersensitive to cell wall stress (Fig 7). We posit that this phenotype is based in their perturbed carbon source metabolism and inefficient respiration (Figs 3B and 4) together with their dysregulated phosphate homeostasis (Figs 2C and 10B). These factors together lead to inadequate ATP supply for biosynthesis of cell wall polysaccharide monomers, i.e. nucleotide sugars [88]; we previously noted defective cell wall construction in *C*. *albicans* cells with inadequate phosphate supplies [88]. Additionally, transcriptional induction of cell wall polysaccharide biosynthesis was deficient in Del381 cells (S2 File). If loss of Tor1 N-terminal HEAT repeats disturbs the conformation of Kog1 with which the N-HEAT domain is known to physically interact, specific phenotypes we observed could also be due to faulty Snf1 signaling, as recently described by Laxman and colleagues [109], who found that Kog1 controls Snf1 in *S*. *cerevisiae* independently of Tor kinase activity. When the conformation of Kog1 is disturbed by an N-terminal epitope tag, cells have specific growth defects only in synthetic media with non-fermentable carbon sources and ammonium as the sole nitrogen source. They are defective in apportioning intermediate metabolites to gluconeogenesis and instead skew them toward amino acid biosynthesis [109]. Whether loss of N-terminal HEAT repeats affects Snf1 function through distortion of Kog1 conformation remains to be seen. We cannot rule out that stress response circuits between TORC1 and Rho1 are disrupted in Del381 cells by a conformational change of TORC1 at the site of the Kog1 domain bound alternatively by Tap42 phosphatase or by Rho1 [76,110,111]. *S*. *cerevisiae* Rho1 directs responses to heat and other stressors as well as to cell wall stress in this circuit [76], and its perturbation may not necessarily be detectable as defective Mkc1 activation [76].

Del381 cells’ poor growth on non-fermentable carbon sources (Fig 3B), their defect in oxygen consumption (Fig 4A) and their hypersensitivity to inhibitors of specific complexes (Complexes II, III and IV) within the electron transport chain (Fig 4B) indicate involvement of Tor1 N-terminal HEAT repeats in mitochondrial function, as shown years ago for mTOR [112,113]. These findings are compatible with a role of this region in regulating NADH dehydrogenase, Complex I of the electron transport chain, whose chemical inhibition did not further decrease growth of cells overexpressing *TOR1-Del381*. Mutants in *C*. *albicans* Complex I components have previously been shown to be defective in cell wall mannan biosynthesis [102], confirming the connection between functional respiration and adequate cell wall construction and -maintenance. Consistent with this line of reasoning, transcription of Complex I components was reduced and that of carbon source metabolism genes was perturbed in Del381 cells (Fig 12A and 12C). Intolerance of nutritional stress during unavailability of fermentable carbon sources in the host, and its connection to intolerance of cell wall stress, add to the predicted disadvantages of cells lacking Tor1 N-terminal HEAT repeats’ function during the host interaction.

Further work will be needed to analyze the role of Tor1 N-terminal HEAT repeats during transcription from the native promoter, and to identify the specific protein partners with which they interact for the regulatory functions we elucidated here. We focused on TORC1 though truncation of Tor1 is likely to also affect its role in the rapamycin-insensitive TOR complex 2 (TORC2) [114]. The prominence of inappropriately active anabolic processes in Del381 cells during oxidative stress, like ribosome biosynthesis and translation, indicates that the responses we examined act in the context of TORC1. Which TORC2 functions are disrupted by loss of Tor1 N-terminal HEAT repeats will need to be studied in further investigations of this complex’s interaction partners with Tor1.

**Fig 13:**
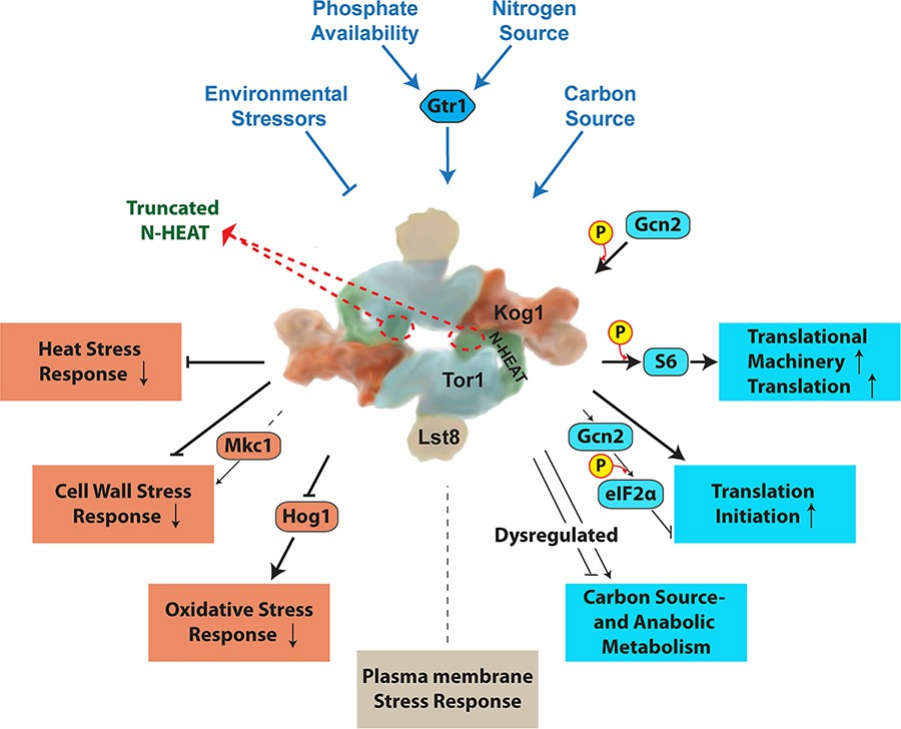
Model of the role of Tor1 N-terminal HEAT repeats in nutritional- and stress responses. Cartoon of TORC1 without Tco89 after PDB:6BCX and [14] with regulatory inputs and outputs examined in this work. Tor1 N-terminal HEAT repeats are shown in green and the remainder of the protein in blue.

Our findings highlight the importance of intact, physiologically modulated TORC1 signaling in specific stress responses critical to *C*. *albicans*’ interaction with the human host (Fig 13). In the experiments we report here, single stressors were examined in isolation; actually during infection, combinations of these stressors act on *C*. *albicans* as often emphasized by Brown and colleagues e.g. in [115]. Suboptimal TORC1 reactivity hence is predicted to impair *C*. *albicans*’ virulence; differences in their TORC1 signaling system were previously discussed as underlying the virulence differences between the frequent pathogen *C*. *albicans* and its close relative, the rarely invasive *C*. *dublinensis* [116]. Our findings also demonstrate that stepwise functional dissection of distinct segments among the large number of interaction domains of the Tor1 kinase is possible. Together with identification of interaction partners for each segment, as well as changes in the phosphoproteome and metabolic shifts during perturbation of distinct segments, the anatomy of the afferent and efferent pathways to each element of the “brain of the cell” can be defined in more detail than has so far been undertaken. Small molecules that disrupt some of these pathways could become “nonimmunosuppressive rapamycin analogs” as envisioned by Cruz et al. [6]. These authors aim to target the conserved Tor kinase domain in a more fungal-selective manner [6]. Our work suggests it might be possible to use the substantial differences between human and fungal TORC1 components including N-terminal HEAT repeats of the Tor kinase itself (Table 1) to target critical cellular functions in *C*. *albicans*, particularly those required during the host interaction, while avoiding toxicity to humans.

## Methods

All experiments were performed in at least 3 biological replicates on different days unless otherwise stated. Graphs, blots and images show a representative experiment of the replicates, except where indicated that data from 2 or 3 biological replicates were combined into a graph.

### Strains and culture conditions

*C*. *albicans* strains used are shown in Table A in S2 Text. Strains were constructed as described in [42], with plasmids shown in Table B in S2 Text and oligonucleotides shown in Table C in S2 Text, using sequences obtained from the Candida Genome Database [117]. To minimize phenotypic artifacts originating from genomic events unrelated to the targeted introduced mutations, all genotypes examined were constructed from at least 2 independently engineered heterozygous strains. *C*. *albicans* cells were grown as described in [42], with addition of 0.3 mM histidine to synthetic media, unless otherwise stated. Anaerobic growth was tested in an Air Lock chamber (Coy Laboratory Products) in N_2_ with 10% H_2_ and 10% CO_2_.

### Growth curves

Stocks stored at -80^o^ were recovered on YPD agar medium for 2 days. Cells were scraped from the plate and washed once in 0.9% NaCl and diluted at OD_600_ 0.01 (for phenotypes for antifungal sensitivity) or OD_600_0.1 (for nutrient phenotypes) in 150 μl medium in flat bottom 96-well dishes. OD_600_ readings were obtained every 15 minutes in a BioTek Synergy 2 Multi-Mode Microplate Reader (Winooski, VT, USA). Standard deviations of 3 technical replicates, representing separate wells, were calculated and graphed in Graphpad Prism Version 9.1.0 (216), and displayed as error bars.

### Western blot

Cell lysis and Western blotting were performed as described in [25]. Antibodies used are listed in Table D S2 Text. For densitometry, ImageJ (imagej.net/welcome) software (opensource) was used to quantitate signals obtained either from KODAK Image Station 4000MM or from Azure biosystems c600.

### Growth of cell dilution spots on solid media

Serial cell dilution spotting assays on agar media was performed as previously described [42]. Briefly, cells recovered from glycerol stock were grown on YPD agar media for 36–48 h, then washed in 0.9% NaCl and diluted with 0.9% NaCl in 5-fold steps from a starting OD_600_ of 0.5 in a microtiter plate, then pin transferred to different agar media. Plates were imaged after 48 hours of incubation at 30^o^ unless stated otherwise.

### Oxygen consumption measurement

Oxygen consumption was measured using a Clark-type electrode (dual digital-model 20; Rank Brothers. Ltd., Cambridge, United Kingdom) at 25^o^, according to the manufacturer’s instructions. *C*. *albicans* cells were grown to mid-logarithmic phase in YPD at 30°, washed twice in 0.9% NaCl and resuspended in the same solution. Cell suspensions were prepared at 5x10^6^ cells/ml. As a control for oxygen saturation, one chamber was filled with 700 μl 0.9% NaCl; in the second chamber 650 μl of cell suspensions was added to 50 μl glucose (to achieve a 2% final glucose concentration). Oxygen saturation was recorded every 3 minutes.

### ROS measurement

Yeast cells grown overnight in YPD for 15 hours were washed twice with 0.9% NaCl and diluted in SC medium (LoFlo) at OD_600_ of 0.5. The fluorescent dye 2’7’-dichlorodihydrofluorescein diacetate (DCFDA) (Sigma, Cat#D6883) was added into the medium to a final concentration of 50 μM. After incubation for 90 minutes, cells were washed twice with 0.9% NaCl and 100 μl per well was placed in a 96-well black plate with optical bottom (Thermo Scientific Nunc), in triplicate. The intensity of fluorescence was read in a BioTek Synergy 2 Multi-Mode Microplate Reader (Winooski, VT, USA) at excitation 485 nm and emission wavelength 528 nm, and a ratio of fluorescence intensity to OD_600_ of the culture was calculated.

### Hyphal morphogenesis assay

Cells were revived from frozen stocks on solid YPD medium for 2 days, washed and resuspended in 0.9% NaCl to OD_600_ of 0.1. Variations between single colonies and colony density effects were minimized by spotting 3 μl cell suspension at 6 equidistant points, using a template, around the perimeter of an agar medium plate. M199, YPD plus 10% serum and Spider medium were used. M199 medium was buffered to pH 5 with 100 mM MES and to pH 7 with 165 mM MOPS. Plates were incubated at 37° between 2 and 4 days. For phenotypes under anaerobic conditions, plates were incubated in an anaerobic chamber (COY lab products) with a mix of 10% Hydrogen, 10% Carbon dioxide and Nitrogen balance at 37°. All panels shown represent at least 2 biological replicates.

### Flocculation

Cells from YPD plates with 10 ng/ml doxycycline were collected and washed once in 0.9% NaCl, then inoculated into YPD medium containing 100 ng/ml doxycycline with a starting OD_600_ of 0.2. Cells were grown at 30^o^ till exponential phase (~4 h). Cells were washed twice in 0.9% NaCl and inoculated into 5.5 ml Spider medium (with additional 0.3 mM histidine) with or without 300 ng/ml doxycycline, with final OD_600_ of 0.7. Cultures were incubated at 37^o^, 200 rpm for 3 h, then settled at room temperature for 15 min before imaging.

### Quantitative real-time PCR analysis

For *tetO*-repression and -derepression, cells were grown in YPD with 2 μg/ml doxycycline with starting OD_600_ of 0.7 for 4 h (Time 0), washed twice with 0.9% NaCl and inoculated into YNB without ammonium sulfate ((NH_4_)_2_SO_4_) supplemented with 10 mM Proline (Pro) as sole nitrogen source, at starting OD_600_ of 0.5, cells were collected at 45 min and 90 min time points. For plumbagin treatment, cells were grown in YPD with 5 ng/ml doxycycline with starting OD_600_ of 0.3 for 3.5 h (Time 0), washed twice in YPD with 5 ng/ml doxycycline and inoculated into YPD with 5 ng/ml doxycycline and 10 μM plumbagin, at starting OD_600_ of 0.4. Cells were collected at 20 min and 40 min time points centrifugation at the indicated time-points, washed 2 times in ice-cold normal saline (0.9% NaCl) and re-suspended in 1 ml cold TRI Reagent (Molecular Research Center Inc, TR118). Total RNA was extracted using Direct-zol RNA Miniprep Plus kit (Zymo Research, R2073) and reverse transcription was performed using qScript cDNA Synthesis Kit (Quanta Bio, 95047–100), both according to the manufacturer instructions. Quantitative real-time PCR was performed with Maxima SYBR Green/ROX qPCR Master Mix (2X) (ThermoFisher, K0222) with *CaTOR1* gene specific primers, using QuantStudio-3 96-well 0.2 ml Block (ThermoFisher). Gene expression levels were measured relative to *C*. *albicans ACT1* expression and q-PCR data were analyzed with QuantStudio.

### Transcript profiling

Transcriptomes of JKC1549 (*tor1/tetO-TOR1*) and JKC1441 (*tor1/tetO-TOR1-Del381*) were compared with those of the heterozygous control JKC1347 (*tor1/TOR1*). Each strain was cultured for 24 h on solid YPD containing 5 ng/ml of doxycycline. Cells were harvested from plates, washed twice in phosphate buffered saline and inoculated into 50 ml of YPD to an OD_600_ of 0.1 in the presence of doxycycline (30 μg/ml) and cells were harvested for RNA extraction after 2 h and 8 h growth at 37°C with shaking at 200 rpm. Experiments comparing Del381 and the *tor1/TOR1* heterozygote without doxycycline were also performed using identical conditions. Cell pellets were flash frozen in liquid N_2_ and RNA extraction was performed using the Qiagen RNeasy mini-kit as described [118]. The microarrays used in this study were designed from assembly 21 of the *C*. *albicans* genome using eArray from Agilent Technologies (design ID 017942) [119]. A total of 6,101 genes (including 12 mitochondrial genes) are represented by two sets of probes, both spotted in duplicate. Total RNA (100 ng) was labelled with Cy5 or Cy3 using the Two-Color Low Input Quick Amp labelling Kit (Agilent Technologies) according to the manufacturer’s instructions. Array hybridization, washing, scanning and data extraction was carried out as described by Haran et al. [118]. Each condition was examined with four biological replicates including dye swapped replicates. Data for each feature was background corrected and expression normalized using the Lowess transformation method in GeneSpring GX13 (Agilent Technologies). Differential expression between pairs of conditions was examined by comparing relative expression ratios (Log2 values) among genes that satisfied a post-hoc test (Storey with Bootstrapping) with a corrected Q value ≤0.05. Gene set enrichment analysis (GSEA) was carried out as described by Uwamahoro et al. [86] and Haran et al. [118]. The full data set can be accessed at the NCBI GEO repository (Accession GSE182186).

To compare the transcriptional responses of JKC1361 (wild-type), JKC1549 (*tor1/tetO-TOR1*) and JKC1441 (*tor1/tetO-TOR1-Del381*) to plumbagin, cells were grown as above and used to inoculate 200 ml YPD supplemented with 5 ng/ml doxycycline at OD_600_ of 0.3. These cells were grown for 3.5 hours, harvested and used to inoculate a fresh 50 ml YPD at OD_600_ of 0.4. These cultures were treated with plumbagin (10 μM) and incubated at 30° at 200 rpm and RNA was harvested at 30 min and 60 min incubation, as described above. A wild-type JKC1361 culture was also treated with DMSO alone (50 μl) in a control experiment. Three biological replicates were generated for each time point and RNA samples were pooled for cDNA sequencing Using Oxford Nanopore Technologies (Oxford Nanopore Technologies, Oxfordshire, UK) PCR-cDNA Sequencing Kit (SQK-PCS108) and barcoding kit (SQK-LWB001) according to the manufacturer’s instructions. Briefly, total RNA (50 ng) was reverse transcribed to cDNA using the PCR-cDNA Sequencing Kit (Oxford Nanopore Technologies) and SuperScript IV reverse transcriptase (Carlsbad, California, United States) according to the Oxford Nanopore Technologies protocol (version PCB_9037_v108_revF_30Jun2017). Following this, cDNA was amplified and indexed using the PCR Barcoding Kit (Oxford Nanopore Technologies) and LongAmp Taq 2X master mix (New England Biolabs). The resulting cDNA libraries were sequenced on a MinION (MIN101B) device with a FLO-min106 SpotON R9.4 Flowcell using the MinKNOWN software v1.7.10 (Oxford Nanopore Technologies). Fast5 reads that passed filtering where basecalled and demultiplexed using the Albacore software v2.2.3 (Oxford Nanopore Technologies). Porechop v0.2.4 (https://github.com/rrwick/Porechop) was used to further trim reads and a total of 5.16 million reads were obtained. Sequence reads were aligned to the SC5314 genome (Assembly 21) using BWA-MEM and the resulting SAM files were analysed in Strand NGS (Strand Life Sciences). Differential gene expression between pairs of conditions was examined using genes that satisfied a post-hoc test (Storey with Bootstrapping; Q ≤0.05) using GSEA [118]. Raw data can be accessed at NCBI (Accession PRJNA753651).

### Statistical analysis

For growth curves, the total area under the curve and its standard deviation were obtained and normalized based on the mean area for the control group, within a 95% confidence interval, using XY analysis in Prism 9 GraphPad (GraphPad Software, Inc., CA, USA). Statistical significance among groups was determined using an unpaired t-test with Welch’s correction, with two-tailed P values.

## Supporting information

S1 Text Supplementary Figures. Fig A. Sequence alignment of fungal and human Tor kinases shows the most divergent regions to lie at the N-terminus. Fig B. Cells containing *tetO-TOR1-FL* and *tetO-TOR1-Del381* alleles appropriately repressed transcription from *tetO* according to their doxycycline exposure, were hypersensitive to caffeine and showed distinct responses to different nitrogen sources. Fig C. Del381 cells’ growth defect relative to wild type was not increased at pH2 on a fermentable- and a non-fermentable carbon source. Fig D. *TOR1* overexpression from *tetO* was not blocked by plumbagin treatment. Fig E. Premature GFP translation initiation in Del381 cells was not correlated to transcription level. Fig F. Del381 cells were hypersensitive to heat- and cell wall stress and maintained cell wall integrity signaling while heat stress did not affect P-S6 intensities. Fig G. Spots of cells with distinct *TOR1* alleles showed specific surface wrinkling phenotypes.
(PDF)

S2 Text Supplementary Text. **Tables**. Table A. *C*. *albicans* strains used in this study. Table B. Plasmids used in this study. Table C. Oligonucleotides used in this study. Table D. Antibodies used in this study.
(PDF)

S1 File Genes showing 1.5 fold expression increase in D381 relative to heterozygous *TOR1/tor1*. (XLSX)

S2 File Genes showing 1.5 fold expression decrease in D381 relative to heterozygous *TOR1/tor1*. (XLSX)

S3 File Genes showing increased expression in D381 following 2h exposure to Doxycycline. (XLSX)

S4 File Genes showing decreased expression in D381 following 2h exposure to Doxycycline. (XLSX)

S5 File Genes showing increased expression in *tetO-TOR1-FL* following 2h exposure to Doxycycline. (XLSX)

S6 File Genes showing decreased expression in *tetO-TOR1-FL* following 2h exposure to Doxycycline. (XLSX)

S7 File Gene expression SC5314 exposed to 10 μM Plumbagin vs SC5314 exposed to DMSO. (XLSX)

S8 File Gene expression in SC5314 vs *tetO-TOR1-D381* in 10 μM Plumbagin. (XLSX)
