# Supplementary material for: Stress- and metabolic responses of Candida albicans require Tor1 kinase N-terminal HEAT repeats
Source: PLoS Pathog. 2022 Jun 10;18(6):e1010089. doi: 10.1371/journal.ppat.1010089 (PMC9223334; doi:10.1371/journal.ppat.1010089)
Supplement: S1 Text — Fig A. Sequence alignment of fungal and human Tor kinases shows the most divergent regions to lie at the N-terminus. Fig B. Cells containing tetO-TOR1-FL and tetO-TOR1-Del381 alleles appropriately repressed transcription from tetO according to their doxycycline exposure, were hypersensitive to caffeine and showed distinct responses to different nitrogen sources. Fig C. Del381 cells’ growth defect relative to wild type was not increased at pH2 on a fermentable- and a non-fermentable carbon source. Fig D. TOR1 overexpression from tetO was not blocked by plumbagin treatment. Fig E. Premature GFP translation initiation in Del381 cells was not correlated to transcription level. Fig F. Del381 cells were hypersensitive to heat- and cell wall stress and maintained cell wall integrity signaling while heat stress did not affect P-S6 intensities. Fig G. Spots of cells with distinct TOR1 alleles showed specific surface wrinkling phenotypes. (PDF) [file ppat.1010089.s001.pdf]

Supplementary Figures

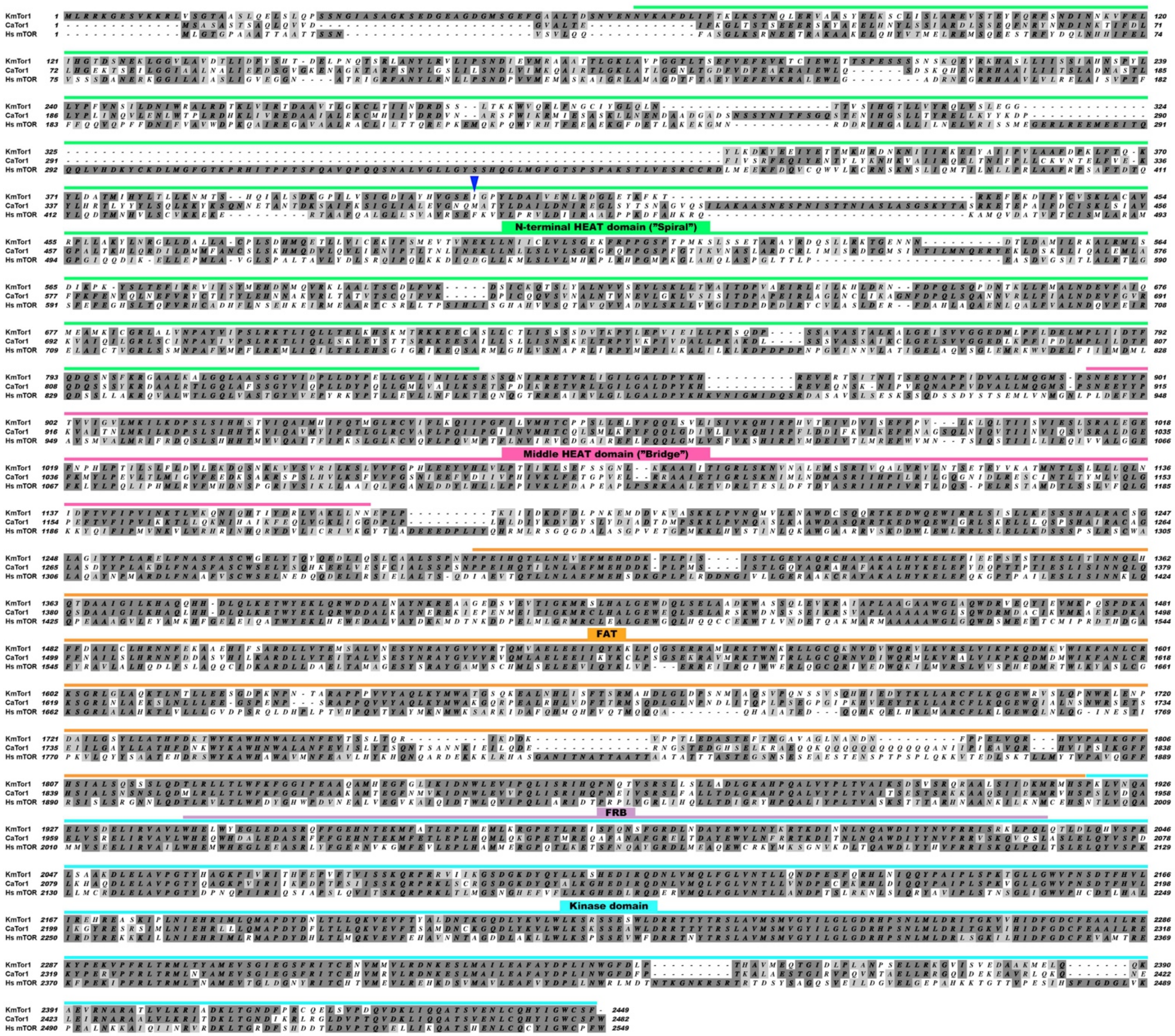

Fig. A. Sequence alignment of fungal and human Tor kinases shows the most divergent regions to lie at the N-terminus. Tor kinase sequences from *Kluyveromyces marxianus* (KmTor1), *Candida albicans* (CaTor1) and human (Hs mTOR) were aligned in MacVector Software using ClustalW (v1.83) multiple sequence alignment. The blue arrow ▼ marks the beginning of the Tor1-Del381 sequence. Domain annotation is adapted from Baretic et al. [18] according to the KmTor1 sequence.

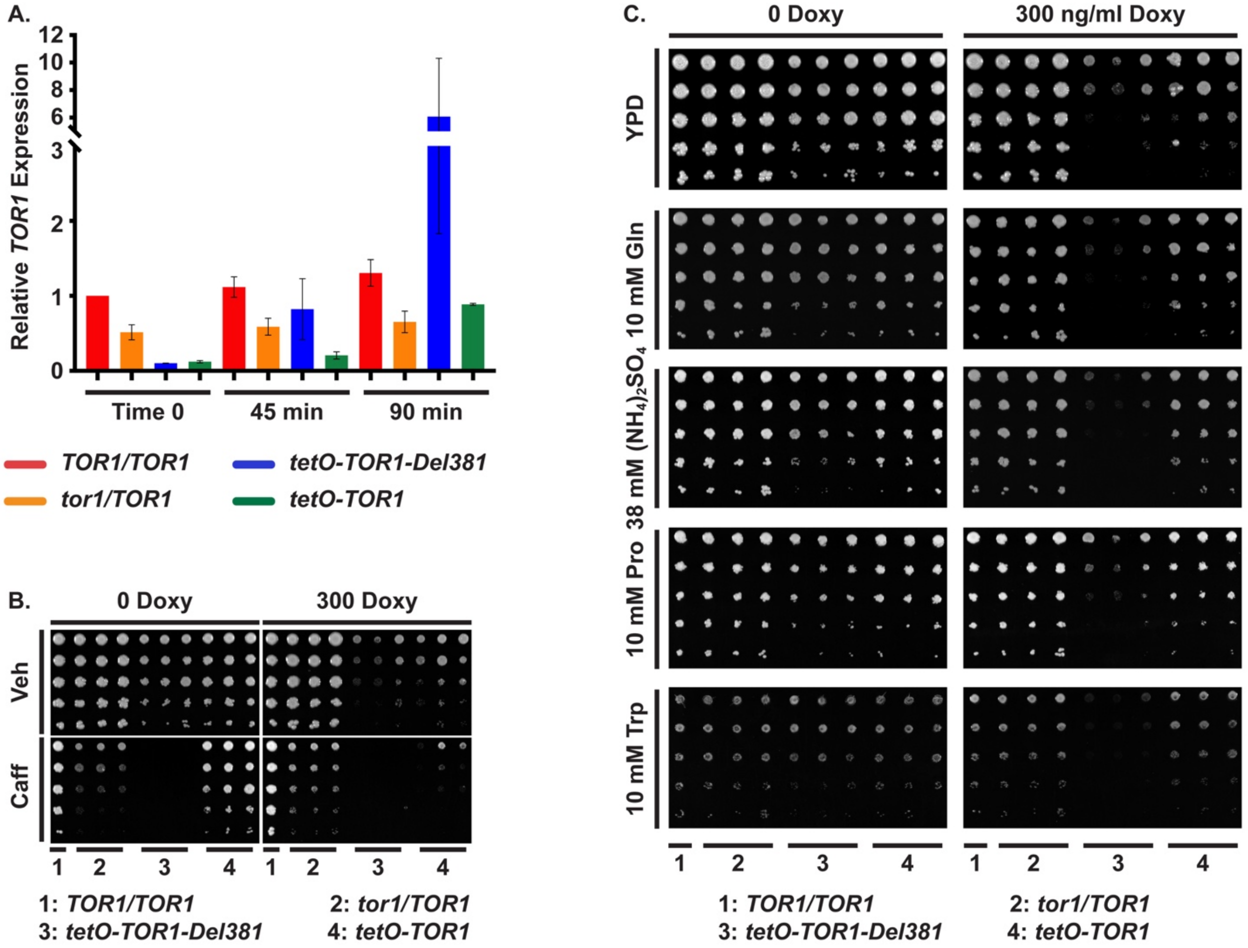

Fig. B. Cells containing *tetO-TOR1-FL* and *tetO-TOR1-Del381* alleles appropriately repressed transcription from *tetO* according to their doxycycline exposure, were hypersensitive to caffeine and showed distinct responses to different nitrogen sources. A. *TOR1* expression from *tetO* rebounded more rapidly in Del381 cells after release from doxycycline repression. Cells of indicated genotypes were pre-grown in YPD medium with 2 µg/ml doxycycline for 4 h, and inoculated into YNB without ammonium sulfate supplemented with 10 mM proline as sole nitrogen source. Cells were harvested at the end of pre-growth (Time 0) and at 45 min and 90 min after inoculation. After qRT-PCR the *TOR1* expression level was measured relative to *ACT1* expression levels. Values are expressed as means ± SD (2 biological replicates using different *TOR1* mutant lineages). (*TOR1/TOR1*, JKC1713; *tor1/TOR1*, JKC1347, JKC1346; *tetO-TOR1-Del381*, JKC1441, JKC1445; *tetO-TOR1,* JKC1549, JKC 1546). **B.** Growth during Caffeine treatment. Dilutions of cells of indicated genotypes were spotted on YPD medium containing either vehicle (Veh, 90% ethanol) or 2.5 mM Caffeine (Caff), without or with 300 ng/ml doxycycline, plates were incubated at 30°C for 2 days. (*TOR1/TOR1*, JKC1713; *tor1/TOR1*, JKC1345, JKC1346, JKC1347; *tetO-TOR1-Del381*, JKC1442, JKC1445, JKC1441; *tetO-TOR1*, JKC1543, JKC1546, JKC1549). **C.** Growth of cell dilutions on media containing different nitrogen sources. Dilutions of cells of indicated genotypes were spotted onto YPD agar medium or YNB agar medium without ammonium sulfate, supplemented with 10 mM glutamine (Gln), 38 mM ammonium sulfate ($(NH_4)_2SO_4$), 10 mM proline (Pro) or 10 mM Tryptophan (Trp) as sole nitrogen source, without or with 300 ng/ml doxycycline (300 Doxy). (*TOR1/TOR1*, JKC1713; *tor1/TOR1*, JKC1345, JKC1346, JKC1347; *tetO-TOR1-Del381*: *tor1/tetO-TOR1-Del381*, JKC1442, JKC1445, JKC1441; *tetO-TOR1*: *tor1/tetO-TOR1-FL,* JKC1543, JKC1546, JKC1549).

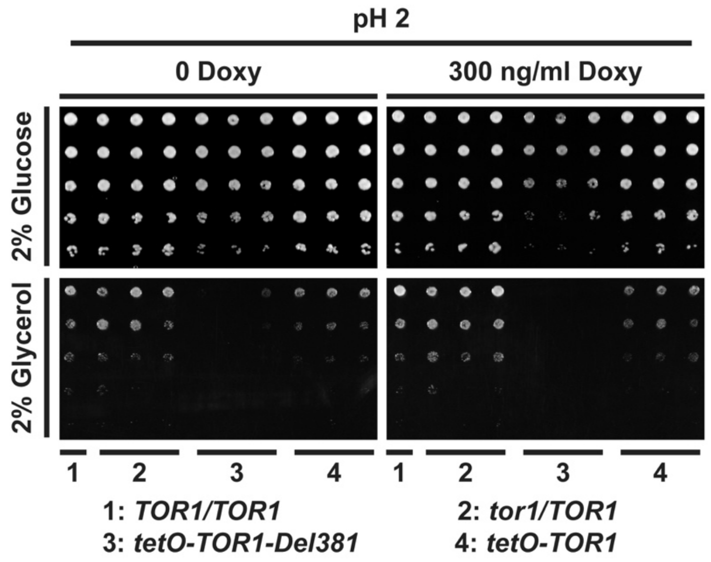

Fig. C. Del381 cells' growth defect relative to wild type was not increased at pH2 on a fermentable- and a non-fermentable carbon source. Cells of indicated genotypes were spotted onto YNB agar medium without inositol, containing glucose or glycerol (2% v/v), without or with 300 ng/ml doxycycline. Medium was buffered to pH2 with 100 mM MES. Representative of 2 biological replicates. (*TOR1/TOR1*, JKC1713; *tor1/TOR1*, JKC1345, JKC1346, JKC1347; *tetO-TOR1-Del381*, JKC1442, JKC1445, JKC1441; *tetO-TOR1,* JKC1543, JKC1546, JKC1549).

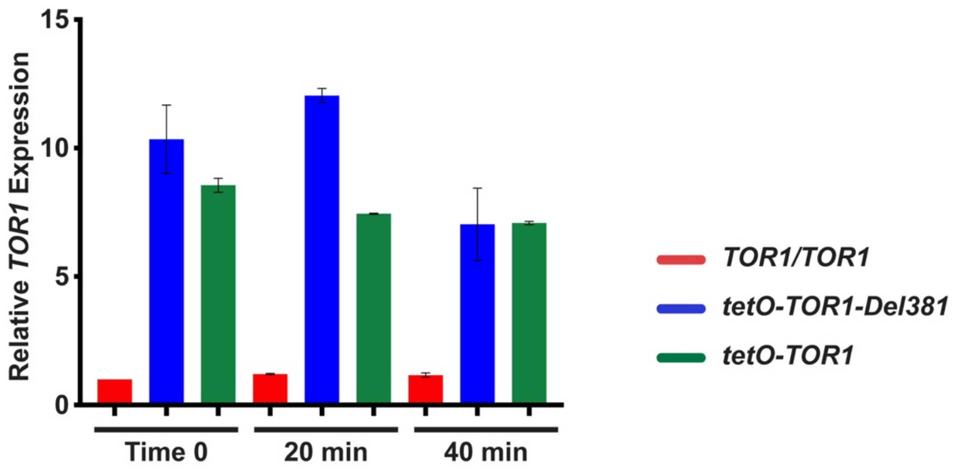

Fig. D. *TOR1* overexpression from *tetO* was not blocked by plumbagin treatment. Cells of indicated genotypes were pre-grown in YPD medium with 5 ng/ml doxycycline for 3.5 h, and inoculated into YPD medium with 5 ng/ml doxycycline and 10 µM plumbagin. Cells were harvested at the end of pre-growth (Time 0) and at 20 min and 40 min after plumbagin exposure. After qRT-PCR, the *TOR1* expression level was assayed relative to *ACT1* expression. Values are expressed as means ± SD (2 biological replicates using different *TOR1* mutant lineages). (*TOR1/TOR1*, JKC1713; *tor1/TOR1*, JKC1347, JKC1346; *tetO-TOR1-Del381*, JKC1441, JKC1445; *tetO-TOR1,* JKC1549, JKC 1546).

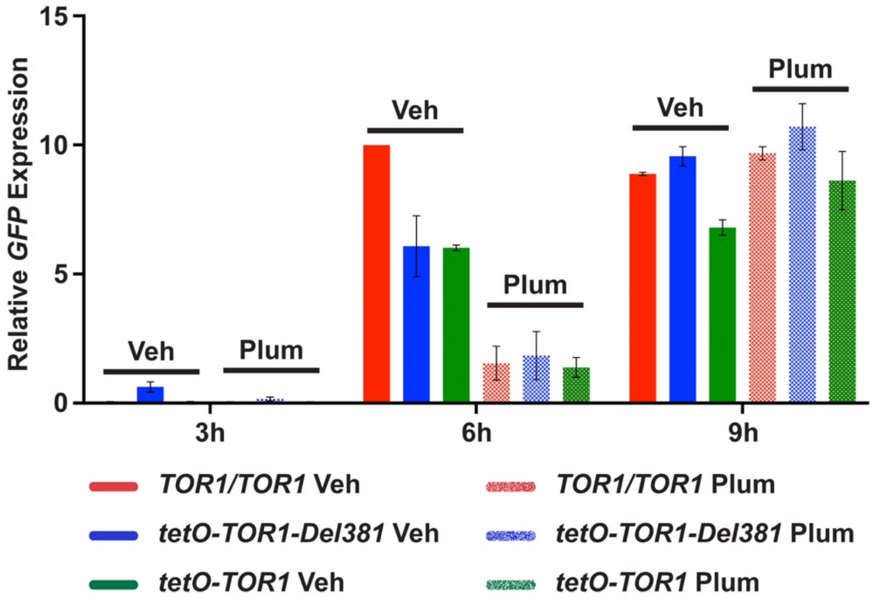

Fig. E. Premature GFP translation initiation in Del381 cells was not correlated to transcription level. Cells expressing *pMAL2*-GFP in backgrounds with distinct *TOR1* alleles were pre-grown in YPD medium with 10 ng/ml doxycycline for 15 h (*TOR1/TOR1*, JKC2616; *tor1/TOR1*, JKC2620; *tetO-TOR1-Del381*, JKC2624; *tetO-TOR1,* JKC2628). GFP expression was induced by inoculation into YP-Maltose medium with 5 ng/ml doxycycline, containing 13 µM Plumbagin (Plum) or DMSO as vehicle (Veh). Cells were harvested at 3 h, 6 h and 9 h after plumbagin or DMSO treatment. After qRT-PCR, the *TOR1* expression level was assayed relative to *ACT1* expression. Values are expressed as means ± SD (2 biological replicates).

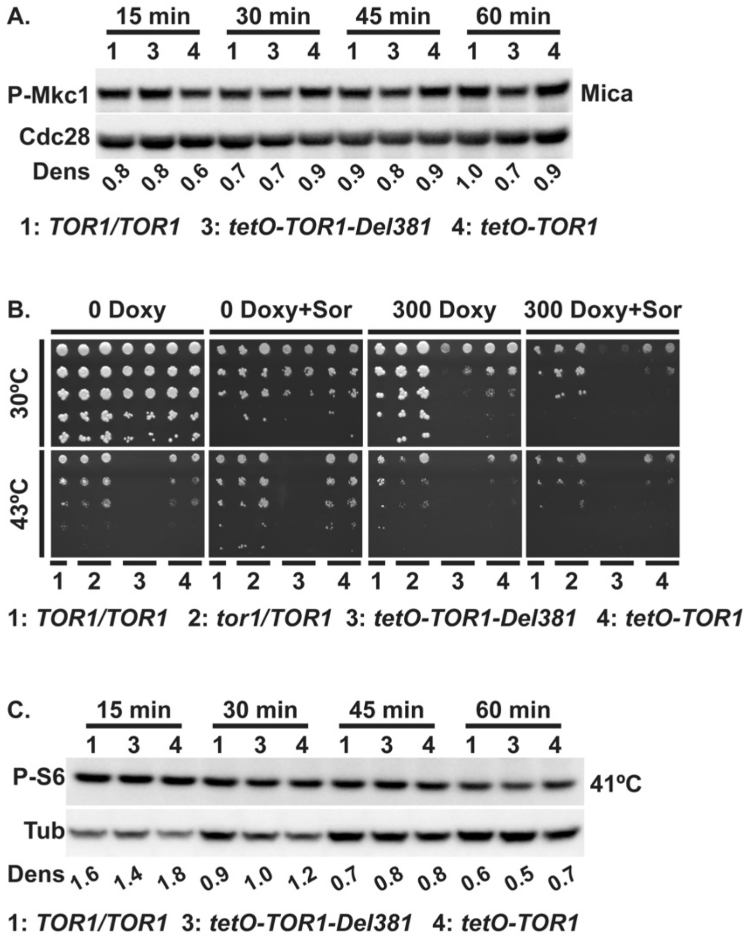

Fig. F. Del381 cells were hypersensitive to heat- and cell wall stress and maintained cell wall integrity signaling while heat stress did not affect P-S6 intensities. A. Mkc1 phosphorylation during cell wall stress. Cells of indicated genotypes were pre-grown in YPD medium with 5 ng/ml doxycycline for 3.5 h and inoculated into YPD medium with 5 ng/ml doxycycline and 10 ng/ml micafungin (Mica). Protein extract was probed with antibody to phosphorylated Mkc1 (P-Mkc1) and the PSTAIRE antigen of Cdc28 as loading control. Dens: signal intensity ratio of P-Mkc1 to Cdc28. Representative of 2 biological replicates. (*TOR1/TOR1*, JKC1713; *tor1/TOR1*, JKC1347; *tetO-TOR1-Del381*, JKC1441; *tetO-TOR1,* JKC1549). **B.** Growth during heat stress. Dilutions of cells of indicated genotypes were spotted on YPD medium without or with 300 ng/ml doxycycline (300 Doxy) and 1M Sorbitol (Sor) and plates were incubated at 30°C or 43°C. (*TOR1/TOR1*, JKC1361; *tor1/TOR1*, JKC1347, JKC1346; *tetO-TOR1-Del381*, JKC1441, JKC1445; *tetO-TOR1*: *tor1/tetO-TOR1-FL,* JKC1549, JKC 1546). **C.** Rps6 phosphorylation during heat stress. Cells of indicated genotypes were pre-grown in YPD medium with 5 ng/ml doxycycline for 3.5 h, inoculated into YPD medium with 5 ng/ml doxycycline and incubated at 41°C for indicated time periods. Total protein extract was probed with antibody to phosphorylated S6 (P-S6), and tubulin (Tub) as loading control. Dens: signal intensity ratio of P-S6 to Tub. Representative of 2 biological replicates. (*TOR1/TOR1*, JKC1713; *tor1/TOR1*, JKC1347; *tetO-TOR1-Del381*, JKC1441; *tetO-TOR1,* JKC1549).

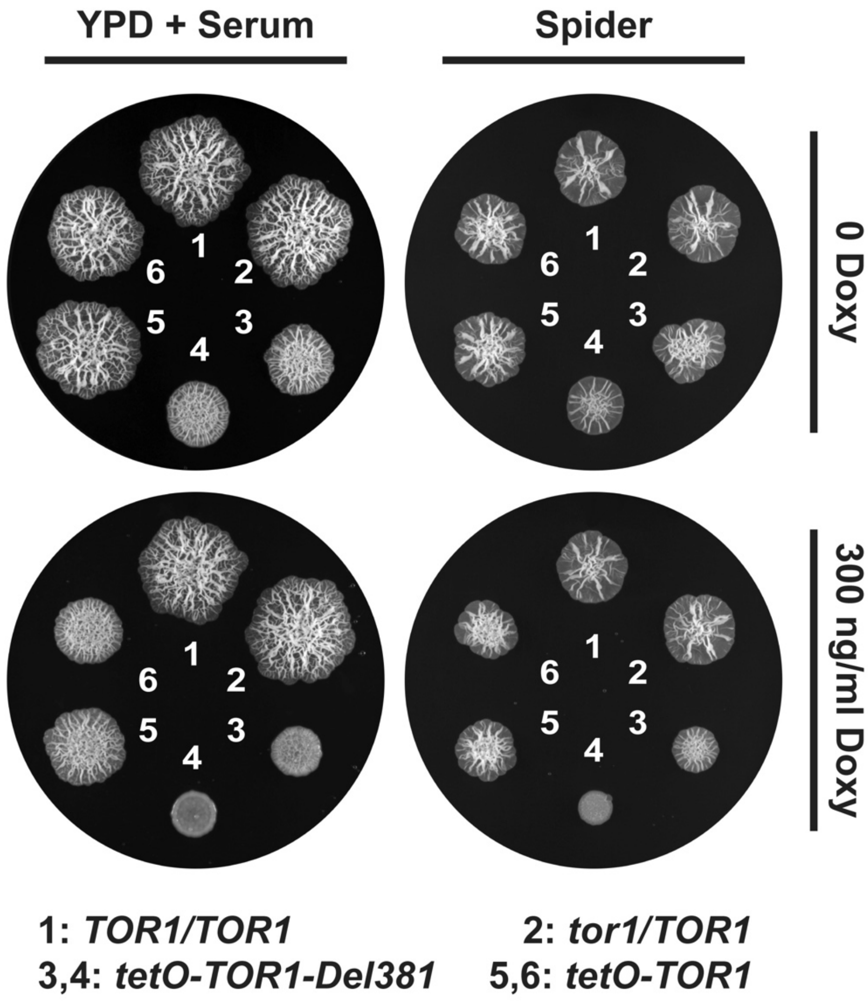

Fig. G. Spots of cells with distinct *TOR1* alleles showed specific surface wrinkling phenotypes. Cells of indicated genotypes were spotted at equidistant points around agar plates, YPD+10% Serum or Spider medium (supplemented with 0.3 mM Histidine, and incubated at 37°C for 3 days, without and with 300 ng/ml Doxycycline. Whole plates were imaged. (*TOR1/TOR1*, JKC1713; *tor1/TOR1*, JKC1347; *tetO-TOR1-Del381*, JKC1441, JKC1445; *tetO-TOR1,* JKC1549, JKC 1546).
