# Supplementary material for: Stress- and metabolic responses of Candida albicans require Tor1 kinase N-terminal HEAT repeats
Source: PLoS Pathog. 2022 Jun 10;18(6):e1010089. doi: 10.1371/journal.ppat.1010089 (PMC9223334; doi:10.1371/journal.ppat.1010089)
Supplement: S2 Text — Tables. Table A. C. albicans strains used in this study. Table B. Plasmids used in this study. Table C. Oligonucleotides used in this study. Table D. Antibodies used in this study. (PDF) [file ppat.1010089.s002.pdf]

Supplementary Text

Tables

Supp. Table Number	Content
**A**	Strains used in this study
**B**	Plasmids used in this study
**C**	Oligos used in this study
**D**	Antibodies used in this study

Table A. *C. albicans* strains used in this study.

C. albicans strain name	Parent	Genotype	Strain background /construction	Reference
SC5314		Wild type		[1]
SN95		*arg4Δ/arg4Δ his1Δ/his1Δ IRO1/iro1Δ::λimm*^{434} *URA3/ura3Δ::λimm*^{434}		[2]
JKC917	SN95	*hisΔ1/his1Δ:: tetR-FRT arg4/arg4 IRO1/iro1Δ::λimm*^{434} *URA3/ura3Δ::λimm*^{434}		[3]
JKC1361	JKC917	*hisΔ1/his1Δ:: tetR-FRT arg4/ARG4 IRO1/iro1Δ::λimm*^{434} *URA3/ura3Δ::λimm*^{434}	JKC917 transformed with PCR product of fjk1184 & rjk1186 using Candida cDNA library as template	[3]
JKC1713	JKC917	*hisΔ1/his1Δ:: tetR-FRT arg4/ARG4 IRO1/iro1Δ::λimm*^{434} *URA3/ura3Δ::λimm*^{434}	JKC917 transformed with PCR product of fjk1184 & rjk1186 using Candida cDNA library as template	This work
JKC1347	JKC917	*tor1::ARG4/TOR1 hisΔ1/his1Δ:: tetR-FRT arg4/arg4 IRO1/iro1Δ::λimm*^{434} *URA3/ura3Δ::λimm*^{434}	JKC917 transformed with PCR product of fjk1142 & rjk1143 using pFA-ARG4 as template	[3]
JKC1345	JKC917	*tor1::ARG4/TOR1 hisΔ1/his1Δ:: tetR-FRT arg4/arg4 IRO1/iro1Δ::λimm*^{434} *URA3/ura3Δ::λimm*^{434}	*tor1/TOR1* heterozygote constructed like JKC1347, independent isolate.	This work
JKC1346	JKC917	*tor1::ARG4/TOR1 hisΔ1/his1Δ:: tetR-FRT arg4/arg4 IRO1/iro1Δ::λimm*^{434} *URA3/ura3Δ::λimm*^{434}	*tor1/TOR1* heterozygote constructed like JKC1347, independent isolate.	This work
JKC1441	JKC1347	*tor1::ARG4/tor1::FRT-tetO-TOR1-Del381 hisΔ1/his1Δ:: tetR-FRT arg4/arg4 IRO1/iro1Δ::λimm*^{434} *URA3/ura3Δ::λimm*^{434}	JKC1347 transformed with StuI/NcoI digested pJK1189 to have *TOR1-Del381* under *tetO (OFF)* promoter, after inducing FLP	[3]
JKC1442	JKC1345	*tor1::ARG4/tor1::FRT-tetO-TOR1-Del381 hisΔ1/his1Δ:: tetR-FRT arg4/arg4 IRO1/iro1Δ::λimm*^{434} *URA3/ura3Δ::λimm*^{434}	*tor1/tetO-TOR1-Del381* constructed like JKC1441, distinct lineage from JKC1345.	This work
JKC1445	JKC1346	*tor1::ARG4/tor1::FRT-tetO-TOR1-Del381 hisΔ1/his1Δ:: tetR-FRT arg4/arg4 IRO1/iro1Δ::λimm*^{434} *URA3/ura3Δ::λimm*^{434}	*tor1/tetO-TOR1-Del381* constructed like JKC1441, distinct lineage from JKC1346.	This work
JKC1549	JKC1347	*tor1::ARG4/tor1::FRT-tetO-TOR1-FL hisΔ1/his1Δ:: tetR-FRT arg4/arg4 IRO1/iro1Δ::λimm*^{434} *URA3/ura3Δ::λimm*^{434}	JKC1347 transformed with StuI/NcoI digested pJK1236 to have *TOR1-FL* under *tetO (OFF)* promoter, after inducing FLP	[3]
JKC1543	JKC1345	*tor1::ARG4/tor1::FRT-tetO-TOR1-FL hisΔ1/his1Δ:: tetR-FRT arg4/arg4 IRO1/iro1Δ::λimm*^{434} *URA3/ura3Δ::λimm*^{434}	*tor1/tetO-TOR1-FL* constructed like JKC1549, distinct lineage from JKC1345.	This work
JKC1546	JKC1346	*tor1::ARG4/tor1::FRT-tetO-TOR1-FL hisΔ1/his1Δ:: tetR-FRT arg4/arg4 IRO1/iro1Δ::λimm*^{434} *URA3/ura3Δ::λimm*^{434}	*tor1/tetO-TOR1-FL* constructed like JKC1549, distinct lineage from JKC1346.	This work
TETG25B	CAI4	*ADH1/adh1::tetO(ON)-GFP*	CAI4 transformed with pTET25 tetracycline inducible GFP cassette	[4]
JKC2616	JKC1713	*hisΔ1/his1Δ:: tetR-FRT arg4/ARG4 MAL2/pMAL2-GFP-FRT-pMAL2-MAL2 IRO1/iro1Δ::λimm*^{434} *URA3/ura3Δ::λimm*^{434}	JKC1713 transformed with BsrGI digested pJK1489 to have GFP under *pMAL2* promoter, after inducing FLP	This work
JKC2620	JKC1347	*tor1::ARG4/tor1::FRT-tetO-TOR1-Del381 hisΔ1/his1Δ:: tetR-FRT arg4/arg4 MAL2/pMAL2-GFP-FRT-pMAL2-MAL2 IRO1/iro1Δ::λimm*^{434} *URA3/ura3Δ::λimm*^{434}	JKC1347 transformed with BsrGI digested pJK1489 to have GFP under *pMAL2* promoter, after inducing FLP	This work
JKC2624	JKC1441	*tor1::ARG4/tor1::FRT-tetO-TOR1-Del381 hisΔ1/his1Δ:: tetR-FRT arg4/arg4 MAL2/pMAL2-GFP-FRT-pMAL2-MAL2 IRO1/iro1Δ::λimm*^{434} *URA3/ura3Δ::λimm*^{434}	JKC1441 transformed with BsrGI digested pJK1489 to have GFP under *pMAL2* promoter, after inducing FLP	This work
JKC2628	JKC1549	*tor1::ARG4/tor1::FRT-tetO-TOR1-FL hisΔ1/his1Δ:: tetR-FRT arg4/arg4 MAL2/pMAL2-GFP-FRT-pMAL2-MAL2 IRO1/iro1Δ::λimm*^{434} *URA3/ura3Δ::λimm*^{434}	JKC1549 transformed with BsrGI digested pJK1489 to have GFP under *pMAL2* promoter, after inducing FLP	This work

Table B. Plasmids used in this study.

Plasmid	Description	Source (Reference)
pJK1000	*FLP-NAT1 tetO-PES1* construct, vector backbone is pLitmus28 (New England Biolabs)	[5]
pJK1027	pAU34 with *URA3* disrupted by $Ag_{promoter}$*TEF1-NAT1-*$Ag_{terminator}$*TEF1*	[6,7]
pJK1189	*FLP-NAT1 tetO-TOR1-Del381* construct, derived from pJK1000.	[3]
pJK1236	*FLP-NAT1 tetO-TOR1-FL* construct, derived from pJK1000.	[3]
pAU15	*pMAL2* expression vector	[6]
pGFP-HIS1	*GFP-HIS1* fusion construct	[8]
pJK1482	*pMAL2-GFP* construct, derived from pAU15. Product of fjk2036 & rjk1633 using pGFP-*HIS1* as template was ligated into pAU15 using SalI/XmaI sites.	This work
pJK1489	*FLP-NAT1 pMAL2-GFP* construct, derived from pJK1482. *URA3* marker in pJK1482 was replaced with the '*FLP-NAT1*' cassette by blunt cloning.	This work

Table C. Oligonucleotides used in this study.

Primer name	Purpose	Sequence 5' to 3' (lower cases - restriction enzyme recognition sites)
fjk2062	Forward primer for *TOR1* quantitative real-time PCR	GCTTAGTTTTATCAGGCAAGGGA
rjk2063	Reverse primer for *TOR1* quantitative real-time PCR	ACTCATCCCCGTGTCTCTAG
fjk1400	Forward primer for *ACT1* quantitative real-time PCR	TGGTGATGGTGTTACTCACG
rjk1401	Reverse primer for *ACT1* quantitative real-time PCR	GACAATTTCTCTTTCAGCAC
fjk1184	Forward primer to amplify the *ARG4* marker	GAATCCACAATCGTATATGAAC
rjk1186	Reverse primer to amplify the *ARG4* marker	GAATATAGTGATGATGAGGATG
fjk1185	Forward primer to confirm the 5'end integration of *ARG4* marker in *Candida* strains.	GACATATTGACCGACATAATTC
rjk1187	Reverse primer to confirm the 5'end integration of *ARG4* marker in *Candida* strains	GTCGTTTCACCGGTGCCACTG
fjk1188	Forward primer to confirm the 3'end integration of *ARG4* marker in *Candida* strains	CAGTACCACCAATAGCATCTC
rjk1199	Reverse primer to confirm the 3'end integration of *ARG4* marker in *Candida* strains	GGTAGTCTCCGATTATGATTC
fjk2036	Forward primer to amplify GFP sequence.	CCTGCTgtcgacATGTCTAAAGGTGAAGAATTAT
rjk1633	Reverse primer to amplify GFP sequence and to confirm the 5'end integration of *pMAL2-GFP* in *Candida* strains	GCAGCTcccgggTTATTTGTATAATTCATCCATACCATGG
fjk2045	Forward primer to confirm the 5'end integration of *pMAL2-GFP* in *Candida* strains	CATTGTGTTGAGCTGCGACT
fjk1517	Forward primer to confirm the 3'end integration of *pMAL2-GFP* in *Candida* strains	GGAATTGTGAGCGGATAAC
rjk2046	Reverse primer to confirm the 3'end integration of *pMAL2-GFP* in *Candida* strains	CAAGGTCCCGTATTTGCTGT

Table D. Antibodies used in this study.

Purpose	Antigen recognized	Species	Source or Reference
primary	P-Mkc1	rabbit	Cell Signaling Technology, #4370P
primary	P-S6	rabbit	Cell Signaling Technology, #9611L
primary	S6	sheep	R&D Systems, #AF5436
primary	P-Hog1	rabbit	Cell Signaling Technology, #4511S
primary	P-eIF2a	rabbit	Cell Signaling Technology, #3597S
primary	GFP	mouse	Roche, #11814460001
loading control	PSTAIRE (Cdc2)	rabbit	Santa Cruz Biotechnology, #sc-53
loading control	Tubulin	rat	Abcam, #ab6161
secondary	Rabbit IgG	goat	Cell Signaling Technology, #7074S
secondary	Sheep IgG	donkey	Santa Cruz Biotechnology, #sc-2473
secondary	Mouse IgG	horse	Cell Signaling Technology, #7076S
secondary	Rat IgG	goat	Abcam, #ab97057

Reference:

1. Fonzi WQ & Irwin MY. Isogenic strain construction and gene mapping in Candida albicans. Genetics. 1993;134(3):717-728.
2. Noble SM & Johnson AD. Strains and strategies for large-scale gene deletion studies of the diploid human fungal pathogen Candida albicans. Eukaryotic Cell. 2005;4(2):298-309.
3. Liu NN, Flanagan PR, Zeng J, Jani NM, Cardenas ME, Moran GP, et al. Phosphate is the third nutrient monitored by TOR in Candida albicans and provides a target for fungal-specific indirect TOR inhibition. Proc Natl Acad Sci U S A. 2017;114(24):6346-51. doi: 10.1073/pnas.1617799114. PubMed PMID: 28566496; PubMed Central PMCID: PMCPMC5474788.
4. Park YN & Morschhäuser J. Tetracycline-inducible gene expression and gene deletion in Candida albicans. Eukaryotic Cell. 2005;4(8):1328-1342.
5. Shen J, Cowen LE, Griffin AM, Chan L, Köhler JR. The Candida albicans pescadillo homolog is required for normal hypha-to-yeast morphogenesis and yeast proliferation. Proc Natl Acad Sci U S A. 2008;105(52):20918-23. Epub 2008/12/17. doi: 10.1073/pnas.0809147105. PubMed PMID: 19075239; PubMed Central PMCID: PMC2634893.
6. Uhl MA & Johnson AD. Development of Streptococcus thermophilus lacZ as a reporter gene for Candida albicans. Microbiology. 2001;147(Pt 5):1189-1195.
7. Patenaude C, Zhang Y, Cormack B, Köhler J, Rao R. Essential role for vacuolar acidification in Candida albicans virulence. The Journal of Biological Chemistry. 2013;288(36):26256-26264.
8. Gerami-Nejad M, Berman J, Gale CA. Cassettes for PCR-mediated construction of green, yellow, and cyan fluorescent protein fusions in Candida albicans. Yeast. 2001;18(9):859-864.
